# Multicomponent Molecular Systems Based on Porphyrins, 1,3,5-Triazine and Carboranes: Synthesis and Characterization

**DOI:** 10.3390/molecules27196200

**Published:** 2022-09-21

**Authors:** Victoria M. Alpatova, Evgeny G. Rys, Elena G. Kononova, Ekaterina A. Khakina, Alina A. Markova, Anna V. Shibaeva, Vladimir A. Kuzmin, Valentina A. Ol’shevskaya

**Affiliations:** 1 A.N. Nesmeyanov Institute of Organoelement Compounds, Russian Academy of Sciences, 28, bld. 1, Vavilova St., 119334 Moscow, Russia; 2 Emanuel Institute of Biochemical Physics, Russian Academy of Sciences, 4 Kosygina St., 119334 Moscow, Russia

**Keywords:** porphyrin, carborane, *s*-triazine, synthesis, phototoxicity, cell death

## Abstract

2,4,6-Trichloro-1,3,5-triazine (cyanuric chloride) is an excellent coupling reagent for the preparation of highly structured multifunctional molecules. Three component systems based on porphyrin, cyanuric chloride and carborane clusters were prepared by a one-pot stepwise amination of cyanuric chloride with 5-(4-aminophenyl)-10,15,20-triphenylporphyrin, followed by replacement of the remaining chlorine atoms with carborane *S*- or *N*-nucleophiles. Some variants of 1,3,5-triazine derivatives containing porphyrin, carborane and residues of biologically active compounds such as maleimide, glycine methyl ester as well as thioglycolic acid, mercaptoethanol and hexafluoroisopropanol were also prepared. A careful control of the reaction temperature during the substitution reactions will allow the synthesis of desired compounds in a good to high yields. The structures of synthesized compounds were determined with UV-vis, IR, ^1^H NMR, ^11^B NMR, MALDI-TOF or LC-MS spectroscopic data. The dark and photocytotoxicity as well as intracellular localization and photoinduced cell death for compounds **8**, **9**, **17**, **18** and **24** were evaluated.

## 1. Introduction

Natural and synthetic porphyrins due to their unique photophysical and photochemical properties have a great potential as systems for the design of molecular electronics and photonics devices [1,2], sensors [3,4], efficient catalysts [5,6], dyes for photovoltaic solar cells [7,8,9] and as photosensitisers (PS) for photodynamic therapy (PDT) against malignant and non-malignant diseases [10,11,12]. The intensive use of porphyrin derivatives in PDT is based on their special physico-chemical and structural properties, such as intense absorption and emission in the visible region where the biological tissues absorb only weakly, high triplet state quantum yield, selectivity for tumor cells and low in vivo toxicity. PDT is currently used as an alternative treatment for the control of malignant diseases [13,14,15] and relies on the combination of a non-toxic PS with harmless visible light to produce reactive oxygen species (ROS), mainly singlet oxygen (^1^O_2_) in target tissues, which can selectively kill cancer cells [16]. Due to that, PDT treatment is safe and has few side effects. Now, PDT is used in the clinic to treat various cancers including tumors of the skin, head, neck and lung [17]. In addition to PDT, other promising modalities for biomedical porphyrins applications as PS have been developed over recent years, such as photodynamic antimicrobial chemotherapy [18,19,20], photothermal therapy [21,22] and sonotherapy [23]. Recently, potential of their application as PS was extended for the treatment of other diseases, such as age-related macular degeneration, skin disorders [24,25,26] and for the inactivation of microorganisms such as fungi or viruses [27,28,29]. The creation of new porphyrin compounds with particular structure and promising biological activity based on affordable starting materials is of considerable interest and substantially extends the potential of porphyrin applications.

In this context the functionalization of porphyrin macrocycles with biologically active nitrogen-containing heterocycles represents an important approach for the improvement of their PDT efficiency [30]. Now, synthesis of nitrogen-containing heterocyclic compounds has attracted an increased interest because of their utility for various biological receptors with a high degree of binding affinity. Triazine derivatives are attractive compounds with biologically important properties, and they have found a number of applications in medicinal chemistry as anticancer [31,32], antimalarial [33,34,35,36,37,38,39], antiviral [40,41] and antimicrobial [42] agents. A convenient compound for the functionalization of the 1,3,5-triazine core with a variety of pharmacaphore groups is 2,4,6-trichloro-1,3,5-triazine (cyanuric chloride). Furthermore, the three symmetrically positioned electrophilic centers of trichlorotriazine enable an alternative method for the selective introduction of diversity of functionalities through the successive substitution of the chlorine atoms with nucleophiles via the addition–elimination mechanism [43]. In recent years, our studies have focused on the design of boronated porphyrin derivatives for PDT and BNCT containing covalently bound 1,2,3-triazole [30] and maleimide moieties [44] on the periphery of *meso*-tetraarylporphyrins. There is available information on the improved antitumor properties of tetrapyrrole photosensitizers containing boron polyhedra in PDT as compared with their non-boronated analogues [45,46,47]. At the same time, the literature survey indicated that carboranyl-substituted 1,3,5-triazines also demonstrated biological activity [48] and are efficient compounds towards creation BNCT agents. One, two or three carboranyl residues were incorporated into cyanuric chloride [49,50,51,52].

In this study, we report the synthesis of carboranyl-substituted porphyrin conjugates containing 1,3,5-triazine (*s*-triazine) heterocycle as a rigid spacer group by replacing one or two chlorine atoms of cyanuric chloride with different *S*- and *N*-carborane nucleophiles. Therefore, the combining within one molecule porphyrin, *s*-triazine heterocycle and carborane cluster may result in the molecules with new biological profile.

## 2. Results and Discussion

### 2.1. Synthesis

For the synthesis of porphyrin-triazine conjugates containing carborane clusters, readily available 5-(4-aminophenyl)-10,15,20-triphenylporphyrin **1 [53]** and cyanuric chloride **2** were used as starting compounds. Cyanuric chloride **2** is an electron-deficient heterocycle and behaves as a heterocyclic acid chloride analog, in which three of its reactive chlorine atoms are capable of easily being substituted in S_N_Ar reactions. Therefore, it can serve as starting material for the preparation of substituted *s*-triazines, where the substituents are linked via nucleophilic heteroatoms such as oxygen, nitrogen or sulfur [54,55]. This process is temperature-dependent since the reactivity decreases with increasing number of substituents linked to the heterocycle. The structure of the nucleophile, its basic strength and steric factors as well as solvent used for the reaction affected the course of the replacement [56]. Moreover, some examples have been already reported dealing with the preparation of conjugates between the compound **2** and tetrapyrrolic macrocycles such as porphyrins and phthalocyanines [57,58,59,60,61].

**Scheme 1:**
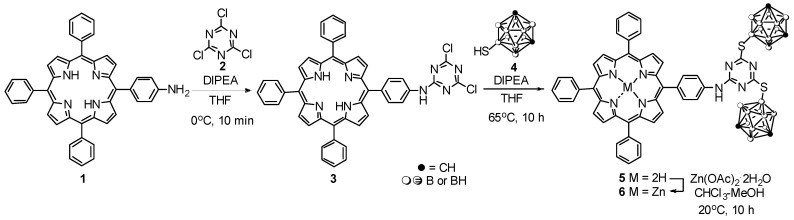
Synthesis of conjugates **5** and **6**.

Porphyrin **1** as *N*-nucleophile was reacted with compound **2** (molar ratio 1:1) at 0 °C in THF in the presence of *N,N*-diisopropylethylamine (DIPEA) to afford conjugate **3**, in which one *s*-triazine chlorine atom was substituted with porphyrin substituent [62]. Its formation was confirmed by disappearance of **1** by TLC analysis in hexane-CHCl_3_ (2:3) system. The reaction was completed in 10 min and, after the separation, conjugate **3** was isolated in excellent (97%) yield. The conjugate **3** was used as key compound in step-wise substitution of the *s*-triazine chlorine atoms with carborane nucleophiles. The conjugate **3** prepared as describe above but without isolation from the reaction mixture was further reacted with an excess of 9-mercapto-*m*-carborane **4** [63] at 65 °C in THF in the presence of DIPEA for 10 h (Scheme 1) to give compound **5** in 82% yield. Treatment of **5** with Zn(OAc)_2_^.^2H_2_O in CHCl_3_-MeOH mixture afforded corresponding zinc complex **6** in 95% yield (Scheme 1).

**Scheme 2:**
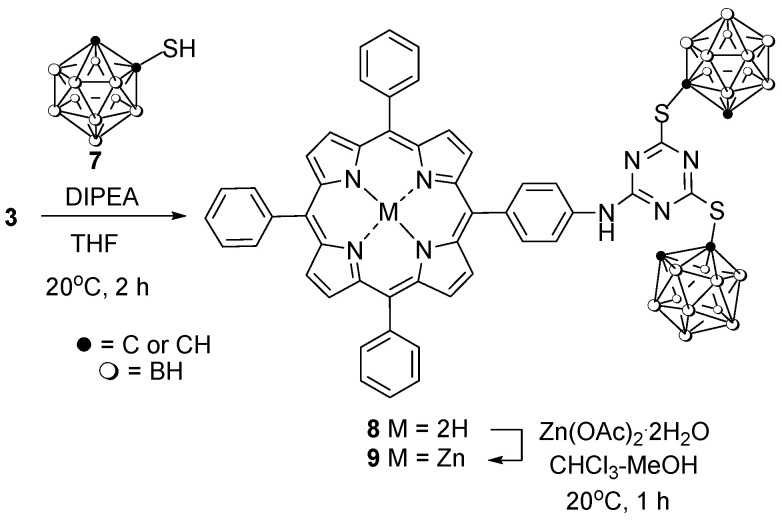
Synthesis of conjugates **8** and **9**.

The reaction of conjugate **3** with 1-mercapto-*o*-carborane **7** [64] containing functional SH-group bound to the carbon atom of carborane polyhedron proceeded smoothly at ambient temperature in THF in the presence of DIPEA for 2 h to afford conjugate **8** in 82% yield. Treatment of **8** with Zn(OAc)_2_^.^2H_2_O in CHCl_3_-MeOH mixture afforded corresponding zinc complex **9** in 94% yield (Scheme 2).

**Scheme 3:**
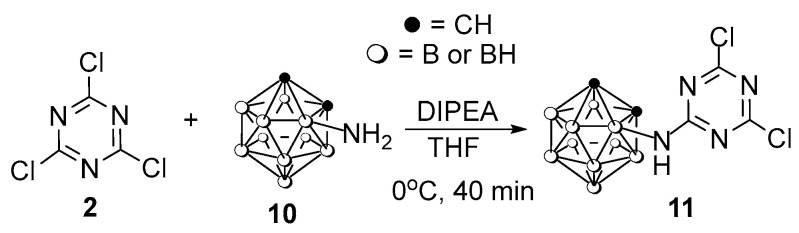
Synthesis of conjugate **11**.

In addition, our attempts to substitute chlorine atoms in **3** with 3-amino-*o*-carborane (**10**) [65] in boiling THF in the presence of DIPEA were unsuccessful. We explain that by the decrease of reactivity of the remaining chlorine atoms in **3 [56]** and the lowered nucleophilicity [66] of amino group in amine **10** in comparison to mercaptocarboranes. At the same time, we have found that reaction of amine **10** with compound **2** was readily proceeded in the presence of DIPEA in THF at 0 °C for 40 min to give carboranylamino dichlorotriazine derivative **11** in 71% yield (Scheme 3).

**Scheme 4:**
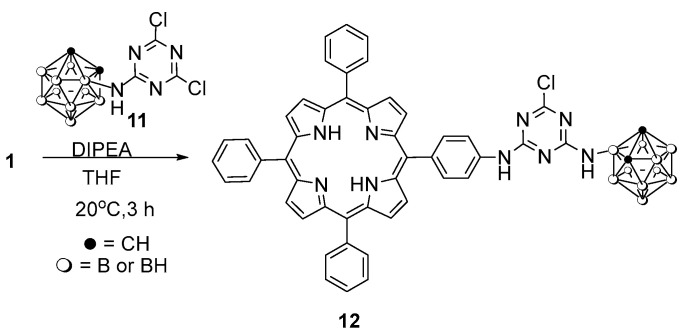
Synthesis of conjugate **12**.

The introduction of porphyrin entity into compound **11** was achieved through the condensation of porphyrin **1** amino group with chlorine atom in compound **11** at ambient temperature for 3 h in THF using DIPEA as an acid scavenger to afford conjugate **12** in 72% yield after the purification by column chromatography (Scheme 4).

**Scheme 5:**
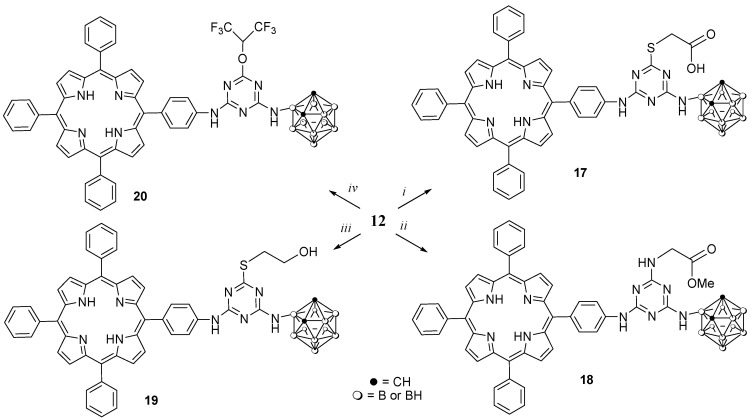
Synthesis of conjugates **17**–**20**: (i) thioglycolic acid (**13**), DIPEA/DMAP, THF, 20 °C, 48 h; (ii) glycine methyl ester hydrochloride (**14**), DIPEA, THF, 65 °C, 24 h; (iii) mercaptoethanol (**15**), DIPEA/DMAP, THF, 65 °C, 6 h; (iv) HFIP (**16**), DIPEA, 65 °C, 5 h.

It turned out that we failed to replace the remaining chlorine atom in conjugate **12** with carborane **10**, but its substitution proceeded easily when reacting with thioglycolic acid (**13**), methyl ester of glycine hydrochloride (**14**), mercaptoethanol (**15**) and hexafluoro-2-propanol (HFIP) (**16**) in THF in the presence of DIPEA or DIPEA/DMAP system in THF to give corresponding derivatives **17**–**20** in rather good yields (Scheme 5). It can be also assumed that simultaneous introduction of substituents with COOH, COOMe, OH and CF_3_ functional groups along with porphyrin and carborane ones in *s*-triazine system may improve the biological characteristics of resulting compounds.

**Scheme 6:**
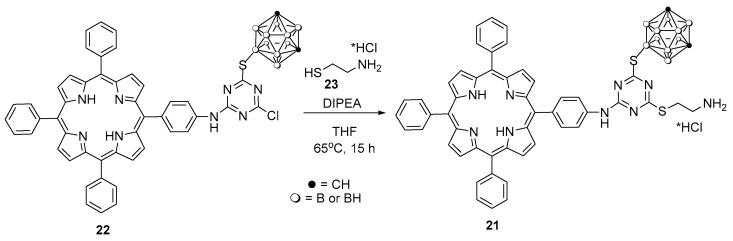
Synthesis of conjugate **21**.

A similar synthetic approach was used for the preparation of trisubstituted *s*-triazine conjugate **21** starting from the disubstituted triazine conjugate **22**, which was formed via the treatment of THF solution of conjugate **3** with 9-mercapto-*m*-carborane (**4**) (1 equiv, DIPEA, 8h). Compound **22** was not isolated but was further converted into the target compound **21** (85%) by refluxing with an excess of 2-aminoethanethiol hydrochloride (**23**) and DIPEA for 15 h (Scheme 6).

**Scheme 7:**
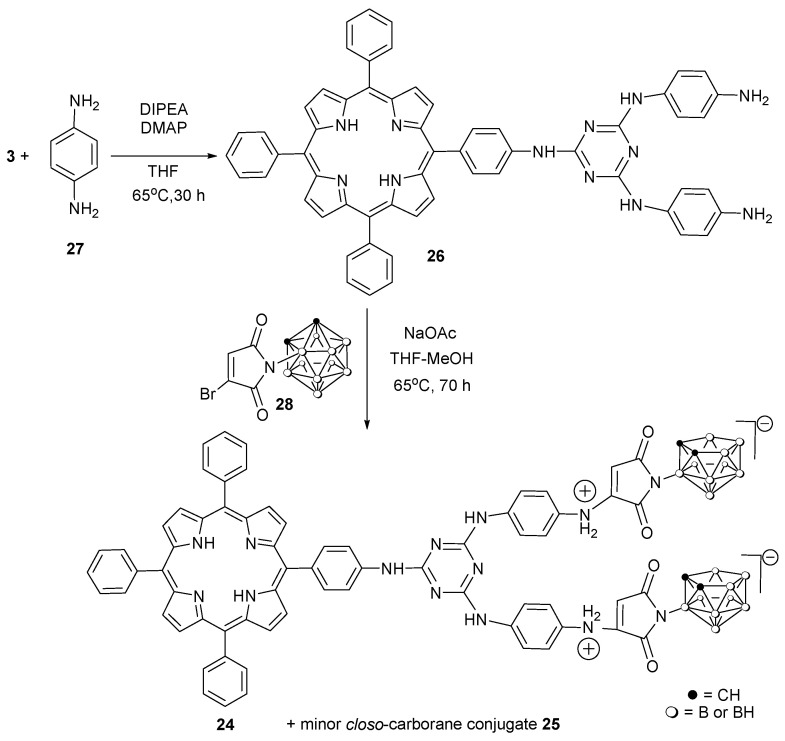
Synthesis of maleimide-substituted conjugates **24**, **25**.

In light of maleimides importance in medicinal chemistry, conjugates **24**, **25** containing a carboranylmaleimide fragments have been obtained by the reaction of phenylenediamine-substituted porphyrin **26** (prepared from conjugate **3** and 1,4-phenylenediamine **27**) with 3-bromo-1-(*N*-(*o*-carborane-3′-yl)maleimide (**28**) [67] (Scheme 7). This reaction resulted in the formation of two products. The major product **24** was zwitter-ionic *nido*-carboranemaleimide-substituted conjugate (89% yield); the minor product was *closo*-carborane conjugate **25** (8% yield).

### 2.2. Spectroscopic Data

All synthesized compounds were isolated by column chromatography and their structures were fully characterized by UV-vis, IR and ^1^H, ^11^B, ^11^B{^1^H} NMR spectroscopies and MALDI or ESI mass spectrometry. IR spectra of boronated porphyrins **5**, **6**, **8**, **9**, **12**, **17**–**21** and **25** contain strong bands for the B–H stretching vibrations in the region of 2587 to 2608 cm^-1^ supporting the carborane *closo*-structure, strong band for the B–H stretching vibrations at 2521 cm^-1^ for porphyrin **24** supporting the carborane *nido*-structure and for N–H stretching vibrations in the region 3269–3418 cm^-1^. The band at 3054–3075 cm^-1^ indicated the presence of the carborane CH groups. Additionally, the IR spectra of all prepared porphyrins **5**, **6**, **8**, **9**, **12**, **17**–**21, 24** and **25** showed the characteristic band in the regions 1521–1596 cm^-1^ corresponding to the C = N bonds of the *s*-triazine heterocycle. In ^1^H NMR eight β-protons of porphyrin macrocycle for all compounds were found between 8.80 and 9.00 ppm and nineteen *meso*-phenyl protons of porphyrins were observed between 7.68 and 8.31 ppm.

The expected signals for functionalities linked at the *s*-triazine spacer were also observed confirming the structure of compounds **5**, **6**, **8**, **9**, **12**, **17**–**21**, **24** and **25** (See Materials and Methods and Supplementary Materials Figures S1–S13 for details).

### 2.3. UV–Visible Absorption and Fluorescence Spectra

**Figure 1:**
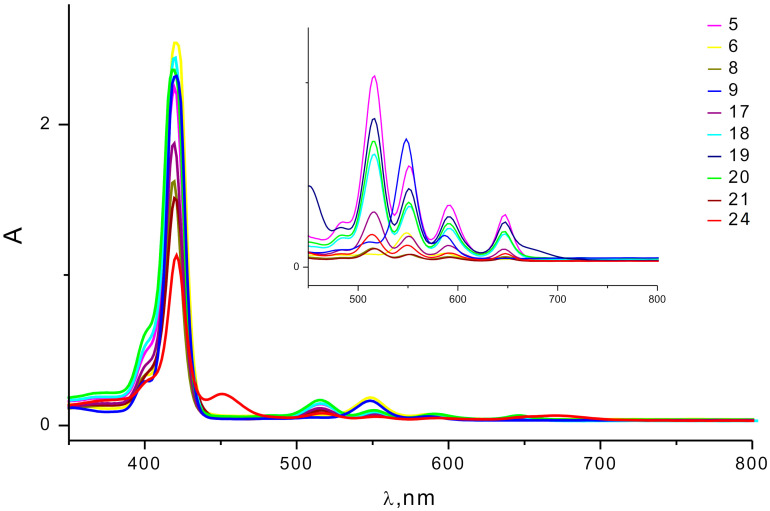
Absorption spectra of compounds **5**, **6**, **8**, **9**, **17**–**21** and **24**.

The UV-vis spectra of newly synthesized compounds **5**, **6**, **8**, **9**, **17**–**21** recorded in CH_2_Cl_2_ and compound **24** recorded in (CH_3_)_2_CO are shown in Figure 1. Most of them demonstrate an intense Soret band between 419 and 421 nm, only for a maleimide-substituted conjugate **24** the corresponding band was found at 416 nm likely due to an increase of HOMO-LUMO energy gap.

Spectra of all the porphyrins exhibit four Q bands between 514 and 647 nm. The intensity pattern of Q bands is of etio-type (IV > III > II > I) in spite of asymmetry of substitution. It reflects the absence of distortion of the π-conjugation of porphyrin core probably due to buffer effect of phenyl groups. In the case of metalloporphyrins (**6** and **9**), there are only two bands of Q type as a result of symmetry enhancement from D_2h_ in porphyrins to D_4h_ in metalloporphyrins.

**Figure 2:**
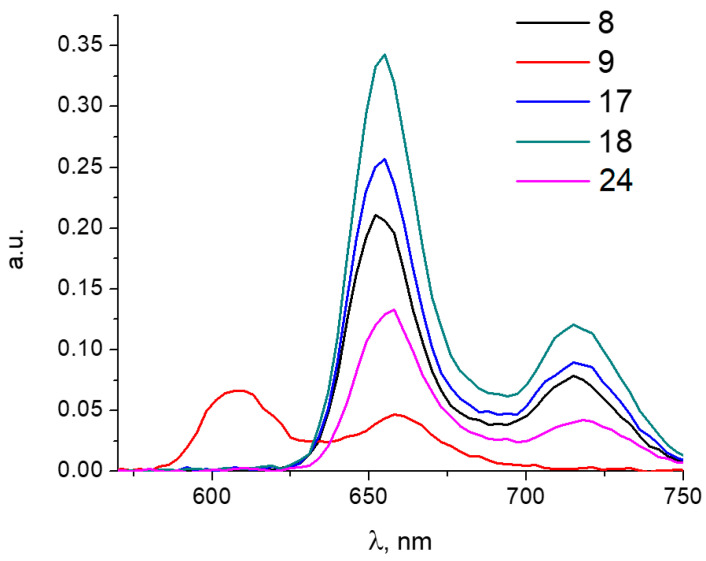
Fluorescence spectra of compounds **8**, **9**, **17**, **18** and **24** in ethanol.

Fluorescence spectra of synthesized compounds **8**, **17**, **18**, **24** (Figure 2) were similar, showing two maxima at 655 nm and 715 nm. The spectrum of porphyrin **9** also showed two fluorescence peaks, with maxima at 610 and 660 nm, which can be explained by the presence of zinc in the molecule.

### 2.4. Cytotoxicity

We evaluated the cytotoxicity of carboranylporphyrins **8**, **9**, **17**, **18** and **24** for tumor cell lines in the dark and after light activation. To determine the dark cytotoxicity, the HCT116 human colon carcinoma cells were exposed to the compounds for 72 h followed by MTT tests. Under these conditions, the cytotoxicity was insignificant: cell survival in the studied range of concentrations was > 75% (For details see Supplementary Materials Figure S14). After photoexcitation with a diode laser at 400 nm, 2 J/cm^2^, cell viability markedly decreased. Upon photoactivation, carboranylporphyrins **9** and **24** were the most potent and caused a dose-dependent cell death after illumination. IC_50_ values were 20 ± 1.8 and 16 ± 0.9 μM, respectively (see Supplementary Materials Figure S15). As positive controls, the disodium salt of chlorin e6 (Photolon) and the fluorinated carboranylchlorin FBCh with the reported PS characteristics were used [68].

Photoexcitation of porphyrins and chlorins in a biological experiment made it possible to compare the obtained results. Photoinduced cytotoxicity data (Figure S15) indicated that the activity dosage form of the clinical PS was somewhat higher, probably due to the fact that the time of intracellular accumulation of prepared carboranylporphyrins **9**, **24** is higher than of Photolon (24 h versus 1.5 h [69], respectively). The activity of compounds **9** and **24** was comparable with FBCh.

Finally, compared to original tetraphenylporphyrin (TPP), the carboranylporphyrins **9** and **24** exhibited an improved phototoxicity in HCT116 cells whereas the dark cytotoxicity was low. Indeed, TPP in the dark did not show cytotoxicity at 0.1–50 μM for 48 h. Upon intracellular accumulation for 24 h, photoexcitation after a higher light exposure (33 J/cm2) followed by 24 h incubation (to allow cell death), TPP showed no photodynamic activity [30].

### 2.5. Intracellular Localization and Photoinduced Cell Death

**Figure 3:**
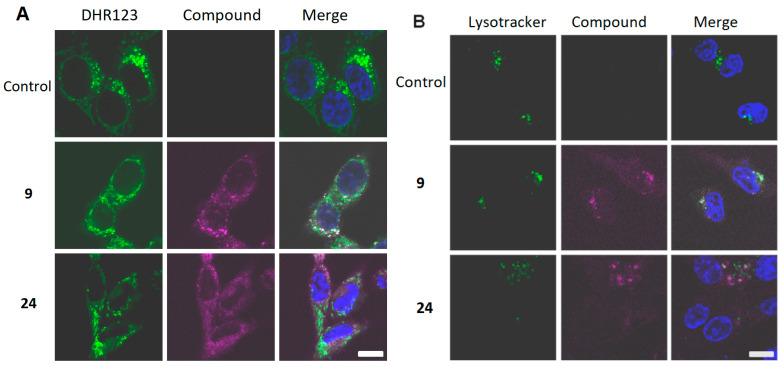
Cytoplasmic distribution of compounds **9** and **24**. The HCT116 cells were loaded with **9** and **24** (10 μM each) for 24 h at 37 °C, 5% CO_2_ in the dark. Colocalization with mitochondria (**A**), lysosomes (**B**). Bar, 15 µm.

Colocalization of carboranylporphyrins **9** and **24** with mitochondrial and lysosomal trackers after 24h of accumulation is shown in Figure 3.

**Figure 4:**
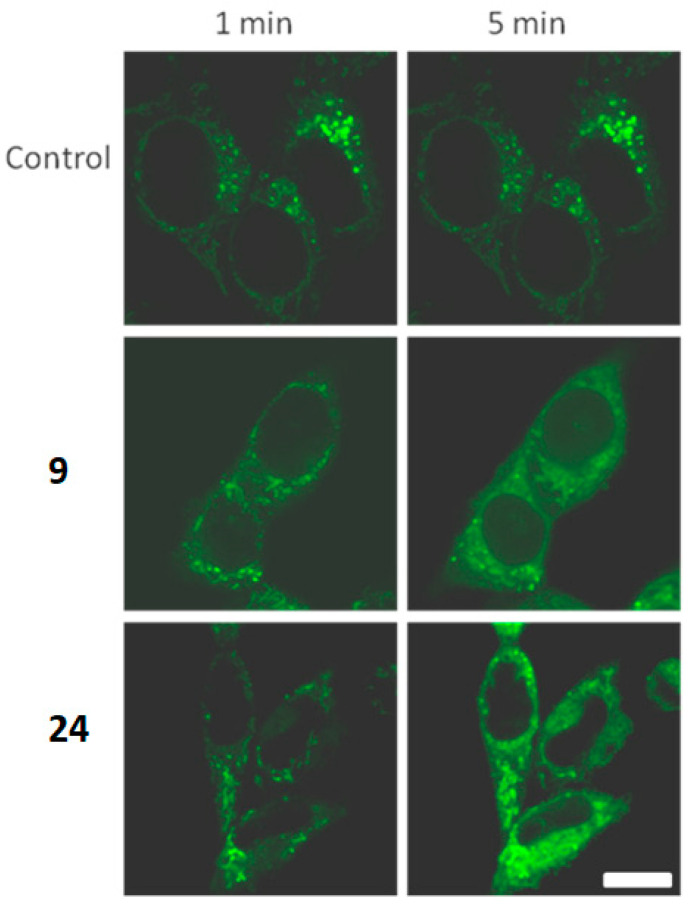
ROS generation after light excitation of photosensitizers accumulated by HCT116 cells for 24 h, final concentration 10 μM. Photoexcitation was carried out with microscope laser for 30 s at 405 nm. Bar, 15 µm.

Both **9** and **24** were distributed in the cytoplasm but not in nuclei. Both compounds colocalized with mitochondria and lysosomes. Compound **9** was distributed as granules. In addition to mitochondria, its colocalization with the lysosomal dye was clearly detectable. For compound **24**, accumulation in lysosomes was less pronounced. During activation with the microscope laser at 405 nm (excitation of Hoechst 33342) an increase in the ROS probe dihydrorhodamine 123 (DHR 123) (Figure 4).

**Figure 5:**
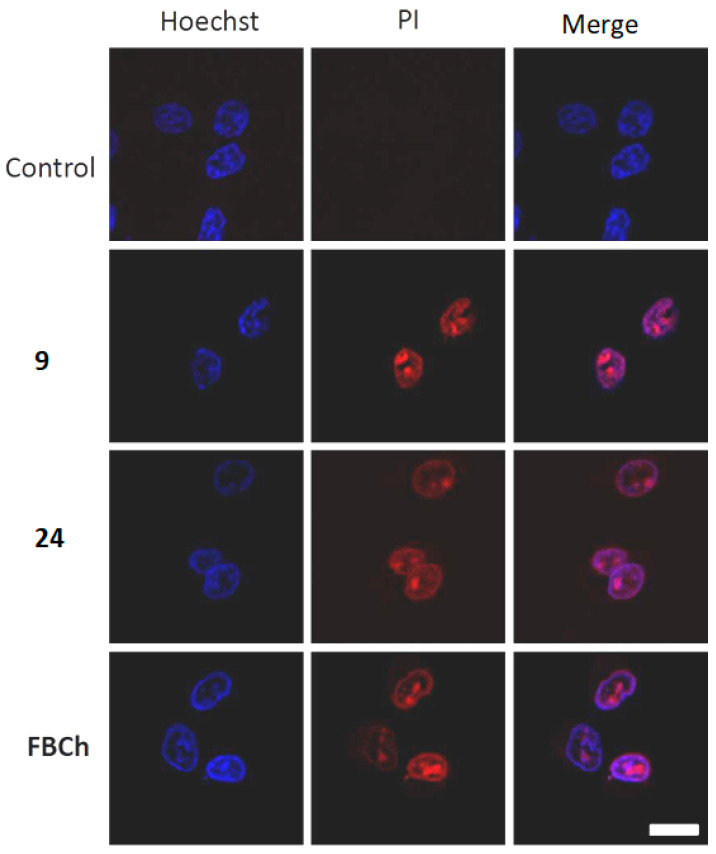
PI entry (necrosis) after light excitation of HCT116 cells loaded with indicated compounds for 24 h (10 μM). Photoexcitation was carried out with the microscope laser for 5 min at 405 nm for **9**, **24** and FBCh. Bar, 15 µm.

ROS generation occurred 5 minutes after photoexcitation of porphyrins **9**, **24** accumulated by the cell, which is an event preceding cell death. To analyze necrotic non-repairable membrane damage under light excitation of photosensitizers, propidium iodide was added to the cell buffer during shooting as a marker of the plasma membrane integrity, and it’s binding to nucleic acids in nucleus made it possible to detect necrotic membrane damage. Compounds **9**, **24** were accumulated cells for 24 h, nuclei were stained with Hoechst 33342 and photosensitizers were photoexcited with a confocal microscope laser at a wavelength of 405 nm in the presence of propidium iodide in the cell buffer (Figure 5).

Propidium iodide (PI) entered the cells (a hallmark of the plasma membrane damage) within the initial minutes of illumination. Both **9** and **24** demonstrated the photodynamic potency similar to the reference photosensitizer FBCh [61]. Thus, the mechanism of cell death upon photoexcitation of lead photosensitizers **9** and **24** is rapid necrosis.

## 3. Materials and Methods

### 3.1. General Information

Reagents were from Sigma-Aldrich unless specified otherwise. All reactions were performed in an atmosphere of dry argon. All solvents were dried as recommended in standard protocols.

^1^H, ^11^B and ^19^F NMR spectra were recorded on a Bruker Avance-400 spectrometer operating at 400.13 MHz for ^1^H NMR, 128.28 MHz for ^11^B NMR, 376.5 MHz for ^19^F NMR. Chemical shifts (δ) were referenced to the residual solvent peak (CDCl_3_ and (CD_3_)_2_CO, ^1^H: 7.26 and 2.05 ppm, respectively) for ^1^H, external BF_3_·OEt_2_ for ^11^B and external CFCl_3_ for ^19^F.

IR spectra were recorded on a Bruker FTIR spectrometer Tensor 37 in KBr pellets.

The UV-vis spectra were measured on a spectrophotometer Carl Zeis Specord M 40 in CH_2_Cl_2_, CHCl_3,_ THF and (CH_3_)_2_CO.

MALDI mass spectra for porphyrins were recorded on a Brucer autoflex speed time-of-flight (TOF) mass obtained mass spectrometer (Bruker Daltonics Inc., Bremen, Germany) equipped with a solid-state ultraviolet (UV) laser of 355 nm (1 kHz repetition rate, 1000 shots for each spectrum) and operated in positive reflectron mode. MALDI mass spectra were recorded by using stainless-steel targets (MTP 384 ground steel; Bruker Daltonics Inc., Germany) containing 384 cells for the deposition of the analyte mixed with matrix; the most intense peaks were given for each compound.

LC-MS (liquid chromatography–mass spectrometry) analysis of the reaction products was performed on Shimadzu LCMS-2020 High Performance Liquid Chromatograph Mass Spectrometer with electrospray ionization (ESI) method and single quadrupole detector (negative and positive ions). Desolvation line/heat block temperature were 250/400 °C. Nitrogen (99.5%) was used like nebulizer and drying gas. Acetonitrile (99.9 + % HPLC gradient grade, Chem-Lab) was used as mobile phase with flow rate 0.8 ml/min without any pretreatment. Compounds were dissolved in acetone, injection volume 30 μL. The mass range between 50 and 2000 was scanned.

Merck silica gel L 0.040–0.080 mesh was used for column chromatography.

The identities of new compounds were verified by TLC on Sorbfil plates.

### 3.2. Synthesis and Analysis of Compounds

5-{4-[[4,6-Bis(*m*-carborane-9′-yl)thio]-(1,3,5)-triazine-2-yl]aminophenyl}-10,15,20-triphenylporphyrin (**5**)

To a solution of cyanuric chloride **2** (12 mg, 0.065 mmol) and DIPEA (13 μL, 0.075 mmol) in THF (4 mL) the solution of porphyrin **1** (40 mg, 0.064 mmol) in THF (8 mL) was added under argon at 0 °C to give porphyrin **3** (TLC control, hexane-CHCl_3_, 2:3), which was used in the reaction without isolation. To the obtained porphyrin **3**, a solution of carborane **4** (72 mg, 0.41 mmol) and DIPEA (78 μL, 0.45 mmol) in THF (3 mL) was added and the mixture was stirred at ambient temperature for 8 h and next was boiled at 65 °C for 10 h until the reaction completion. Then, the mixture was poured into water (100 mL), extracted with CH_2_Cl_2_ and the organic solution was dried over Na_2_SO_4_. After removal of the solvent in vacuo, the residue was purified by column chromatography on silica gel using CH_2_Cl_2_-hexane system (1:1) as an eluent. Yield 55 mg (82%), purple solid. UV-vis (CH_2_Cl_2_) λ_max_/nm (log *ε*): 419 (5.37), 515 (4.04), 551 (3.76), 592 (3.56), 647 (3.49). IR (KBr, cm^−1^) ν_max_: 3317 (NH), 3054 (carborane CH), 2601 (BH), 1567 (C = N). ^1^H NMR (400.1 MHz, CDCl_3_), δ (ppm): 8.97 (br s, 2H, β-H), 8.92 (br s, 6H, β-H), 8.26 (m, 8H, Ph), 8.05 (br s, 2H, Ph), 7.79 (m, 9H, Ph), 2.88 (br s, 4H, carborane CH), −2.69 (br s, 2H, NH). ^11^B NMR (128.3 MHz, CDCl_3_), δ (ppm): 1.5 (br s, 1B, B^9^), −3.7 (br s, 1B, B^9^), −6.2 (d, 4B, *J* = 156 Hz), −9.7 (d, 2B, *J* = 144 Hz), −12.9 (d, 4B, *J* = 140 Hz), −13.9 (d, 4B, *J* = 163 Hz), −17.6 (d, 2B, *J* = 182 Hz), −20.5 (d, 2B, *J* = 175 Hz). MS (MALDI-TOF): *m*/*z* calcd for ‘C_51_H_52_B_20_N_8_S_2′_ [M]^+^ 1060.56; found: 1060.12 [M]^+^.

5-{4-[[4,6-Bis(*m*-carborane-9′-yl)thio]-(1,3,5)-triazine-2-yl]aminophenyl}-10,15,20-triphenylporphyrinato zinc (II) (**6**)

A solution of Zn(OAc)_2_·2H_2_O (33 mg, 0.150 mmol) in MeOH (6 mL) was added to a solution of porphyrin **5** (32 mg, 0.030 mmol) in CHCl_3_ (6 mL). The resulting mixture was stirred for 1 h at room temperature. Then, the reaction mixture was treated with water and extracted with CH_2_Cl_2_ (2 × 10 mL). The organic phase was dried over Na_2_SO_4_, and the solvent was removed under reduced pressure. Yield 32 mg (95%), violet solid. UV-vis (CH_2_Cl_2_) λ_max_/nm (log *ε*): 421 (5.87), 549 (4.46), 588 (3.90). IR (KBr, cm^−1^) ν_max_: 3418 (NH), 3054 (carborane CH), 2608 (BH), 1569 (C=N). ^1^H NMR (400.1 MHz, (CD_3_)_2_CO), δ (ppm): 8.99 (dd, 2H, *J* = 9.5, 4.9 Hz, β-H), 8.89 (m, 6H, β-H), 8.23 (d, 10H, *J* = 7.4 Hz, Ph), 7.78 (d, 9H, *J* = 6.4 Hz, Ph), 2.65 (br s, 2H, carborane CH), 2.62 (br s, 2H, carborane CH). ^11^B NMR (128.3 MHz, (CD_3_)_2_CO), δ (ppm): −2.8 (br s, 1B, B^9^), −3.6 (br s, 1B, B^9^), −5.8 (d, 4B, *J* = 151 Hz), −10.2 (d, 2B, *J* = 149 Hz), −12.7 (d, 4B, *J* = 149 Hz), −13.8 (d, 4B, *J* = 156 Hz), −16.7 (d, 4B, *J* = 168 Hz). MS (MALDI-TOF): *m*/*z* calcd for ‘C_51_H_50_B_20_N_8_S_2_Zn’ [M]^+^ 1120.73; found: 1120.49 [M]^+^.

5-{4-[[4,6-Bis(*o*-carborane-1′-yl)thio]-(1,3,5)-triazine-2-yl]aminophenyl}-10,15,20-triphenylporphyrin (**8**)

To a solution of porphyrin **3** prepared from cyanuric chloride **2** (15 mg, 0.080 mmol), DIPEA (16 μL, 0.092 mmol) and porphyrin **1** (50 mg, 0.080 mmol) in THF (6 mL) in situ as described above for the compound **5** the solution of carborane **7** (42 mg, 0.239 mmol) and DIPEA (48 μL, 0.276 mmol) in THF (2 mL) was added. The mixture was stirred at room temperature under argon for 2 h, then treated with water (100 mL), extracted with CH_2_Cl_2_ (20 mL) and organic solution was dried over Na_2_SO_4_. After removal of the solvent in vacuo, the residue was purified by column chromatography on silica gel using CH_2_Cl_2_-hexane system (1:1) as an eluent to give conjugate **8** (69 mg, yield 82%) as a purple solid. UV-vis (CH_2_Cl_2_) λ_max_/nm (log *ε*): 420 (5.24), 515 (3.83), 550 (3.54), 590 (3.36), 646 (3.28). IR (KBr, cm^−1^) ν_max_: 3327 (NH), 3058 (carborane CH), 2593 (BH), 1521 (C=N). ^1^H NMR (400.1 MHz, CDCl_3_), δ (ppm): 8.88 (d, 6H, *J* = 8.9 Hz, β-H), 8.83 (d, 2H, *J* = 4.8 Hz, β-H), 8.31 (d, 2H, *J* = 8.3 Hz, Ph), 8.24 (d, 6H, *J* = 6.7 Hz, Ph), 7.79 (m, 11H, Ph), 7.63 (br s, 1H, NH), 4.96 (br s, 1H, carborane CH), 4.78 (br s, 1H, carborane CH), −2.77 (br s, 2H, NH). ^11^B NMR (128.3 MHz, CDCl_3_), δ (ppm): −1.9 (br s, 4B), −11.9 (br s, 16B). MS (MALDI-TOF): *m*/*z* calcd for ‘C_51_H_52_B_20_N_8_S_2′_ [M]^+^ 1060.56; found: 1060.08 [M]^+^.

5-{4-[[4,6-Bis(*o*-carborane-1′-yl)thio]-(1,3,5)-triazine-2-yl]aminophenyl}-10,15,20-triphenylporphyrinato zinc (II) (**9**)

A solution of Zn(OAc)_2_·2H_2_O (20 mg, 0.091 mmol) in MeOH (1 mL) was added to a solution of porphyrin **8** (20 mg, 0.019 mmol) in CHCl_3_ (6 mL). The resulting mixture was stirred for 1 h at room temperature. Then, the reaction mixture was treated with water and extracted with CH_2_Cl_2_ (2 × 10 mL). The organic phase was dried over Na_2_SO_4_, and the solvent was removed under reduced pressure. Yield 20 mg (94%), pinkish violet solid. UV-vis (CH_2_Cl_2_) λ_max_/nm (log *ε*): 420 (5.33), 548 (4.12), 587 (3.50). IR (KBr) ν_max_, cm^-1^: 3407 (NH), 3058 (carborane CH), 2591 (BH), 1523 (C=N). ^1^H NMR (400.1 MHz, CDCl_3_), δ (ppm): 9.00 (d, 6H, *J* = 7.0 Hz, β-H), 8.95 (d, 2H, *J* = 4.1 Hz, β-H), 8.26 (br s, 8H, Ph), 7.79 (br s, 11H, Ph), 4.98 (br s, 1H, carborane CH), 4.82 (br s, 1H, carborane CH). ^11^B NMR (128.3 MHz, CDCl_3_), δ (ppm): −2.2 (br s, 4B), −11.2 (br s, 16B). LC-MS (ESI): *m*/*z* calcd for ‘C_51_H_49_B_20_N_8_S_2_Zn’ [M-H]^−^ 1119.7; found: 1119.5 [M-H]^−^ (negative ion mode).

2-[(*o*-Carborane-3′-yl)amino]-4,6-dichloro-(1,3,5)-triazine (**11**)

To a solution of cyanuric chloride **2** (580 mg, 3.14 mmol) in THF (8 mL) a solution of 3-amino-*o*-carborane (**10**) (500 mg, 3.14 mmol) and DIPEA (0.64 ml, 3.67 mmol) in THF (15 mL) was added under argon at 0 °C. The mixture was stirred at 0 °C for 40 min, then treated with water (200 mL) and extracted with CH_2_Cl_2_ (2 × 30 mL). The organic solution was dried over Na_2_SO_4_, and the volatiles were removed under reduced pressure. The residue was purified by column chromatography on silica gel using CHCl_3_-hexane system (1:1) as an eluent. Yield 686 mg (71%), white solid. M.p. 142 °C. IR (KBr, cm^–1^) ν_max_: 3407 (NH), 3075 (carborane CH), 2595 (BH), 1551 (C = N). ^1^H NMR (400.1 MHz, CDCl_3_), δ (ppm): 6.08 (br s, 1H, NH), 4.46 (br s, 2H, carborane CH), 3.37–1.40 (m, 9H, BH). ^11^B NMR (128.3 MHz, CDCl_3_), δ (ppm): −3.7 (d, 2B, *J* = 149 Hz), −7.2 (br s, 1B, B^3^), −10.3 (d, 1B, *J* = 151 Hz), −12.6 (d, 4B, *J* = 163 Hz), −14.5 (d, 2B, *J* = 151 Hz). Anal. calcd. for C_5_H_12_B_10_Cl_2_N_4_: C 19.55, H 3.94, N, 18.24. Found: C 19.71, H 4.08, N 18.22.

5-{4-[[4-(*o*-Carborane-3′-yl)amino]-6-chloro-(1,3,5)-triazine-2-yl]aminophenyl}-10,15,20-triphenylporphyrin (**12**)

To a solution of carborane **11** (110 mg, 0.175 mmol) in THF (3 mL) a solution of porphyrin **2** (111 mg, 0.176 mmol) in THF (10 mL) and DIPEA (38 μL, 0.218 mmol) were added and reaction mixture was stirred for 3h under argon at room temperature. Then the reaction was quenched with water (150 mL) and extracted with CH_2_Cl_2_ (2 × 20 mL). The organic phase was washed with water and dried over Na_2_SO_4_. After the removal of the solvent in vacuo, the remaining product was purified by column chromatography on silica gel using CHCl_3_ as an eluent. Yield 114 mg (72%), purple solid. UV-vis (CH_2_Cl_2_) λ_max_/nm (log *ε*): 421 (5.41), 516 (4.11), 552 (3.80), 591 (3.64), 647 (3.54). IR (KBr, cm^−1^) ν_max_: 3407 (NH), 3075 (carborane CH), 2595 (BH), 1551 (C=N). ^1^H NMR (400.1 MHz, CDCl_3_), δ (ppm): 8.88 (br s, 8H, β-H), 8.24 (br s, 8H, Ph), 7.79 (br s, 11H, Ph), 5.83 (br s, 1H, carborane NH), 4.64 (br s, 2H, carborane CH), −2.76 (br s, 2H, NH). ^11^B NMR (128.3 MHz, CDCl_3_), δ (ppm): −5.2 (br s, 3B), −14.2 (br s, 7B). MS (MALDI-TOF): *m*/*z* calcd for ‘C_49_H_43_B_10_ClN_9′_ [M+H]^+^ 902.42; found: 902.48 [M+H]^+^.

5-{4-[[4-(*o*-Carborane-3′-yl)amino]-(6-carboxymethylthio)-(1,3,5)-triazine-2-yl]-aminophenyl}-10,15,20-triphenylporphyrin (**17**)

To a solution of porphyrin **12** (60 mg, 0.067 mmol) in THF (5 mL) thioglycolic acid (**13**) (24 μL, 0.344 mmol), DIPEA (72 μL, 0.413 mmol) and DMAP (20 mg, 0.164 mmol) were added. The resulting mixture was stirred for 48 h at ambient temperature under argon until TLC analysis revealed complete disappearance of starting compound **12** (TLC control, acetone). After that, the reaction was quenched with water (150 mL) and extracted with CHCl_3_ (2×25 mL). The organic phase was washed with water and dried over Na_2_SO_4_. The solvent was removed under reduced pressure. The residue was purified by column chromatography on silica gel using acetone-MeOH (9:1) mixture as an eluent. Yield 56 mg (87%), purple solid. UV-vis (CH_2_Cl_2_) λ_max_/nm (log *ε*): 420 (5.47), 514 (4.07), 550 (3.83), 591 (3.68), 646 (3.60). IR (KBr, cm^−1^) ν_max_: 3315 (NH), 3056 (carborane CH), 2588 (BH), 1715 (C=O), 1551 (C=N). ^1^H NMR (400.1 MHz, CDCl_3_), δ (ppm): 10.67 (br s, 1H, COOH), 8.83 (br s, 8H, β-H), 8.09 (m, 8H, Ph), 7.73 (m, 11H, Ph), 6.36 (br s, 1H, NH), 6.35 (br s, 1H, NH), 4.70 (br s, 2H, carborane CH), 3.83 (br s, 2H, CH_2_), −2.78 (br s, 2H, NH). ^11^B NMR (128.3 MHz, CDCl_3_), δ (ppm): −5.2 (br s, 3B), −14.1 (br s, 7B). LC-MS (ESI): *m*/*z* calcd for ‘C_51_H_45_B_10_N_9_O_2_S’ [M]^−^ 956.1; found: 955.5 [M]^−^ (negative ion mode).

5-{4-[[4-(*o*-Carborane-3′-yl)amino]-(6-methoxycarbonylmethylamino)-(1,3,5)-triazine-2-yl]aminophenyl}-10,15,20-triphenylporphyrin (**18**)

To a solution of porphyrin **12** (35 mg, 0.039 mmol) in THF (5 mL) methyl ester of glycine hydrochloride (**14**) (49 mg, 0.39 mmol) in THF (5 mL) and DIPEA (84 μL, 0.48 mmol) were added and the reaction mixture was boiled for 24 h under argon untill TLC analysis revealed complete disappearance of starting porphyrin **12** (TLC control, CH_2_Cl_2_). After that, the reaction was quenched with water (150 mL) and extracted with CHCl_3_ (2 × 20 mL). The organic phase was washed with water and dried over Na_2_SO_4_. The solvent was removed under reduced pressure. The residue was purified by column chromatography on silica gel using CH_2_Cl_2_-EtOAc (7:3) mixture as an eluent. Yield 29 mg (78%), purple solid. UV-vis (CH_2_Cl_2_) λ_max_/nm (log *ε*): 420 (5.47), 515 (4.16), 551 (3.90), 590 (3.70), 646 (3.63). IR (KBr, cm^−1^) ν_max_: 3315 (NH), 3058 (carborane CH), 2587 (BH), 1743 (C=O), 1575 (C=N), 1212 (COOMe). ^1^H NMR (400.1 MHz, CDCl_3_), δ (ppm): 8.92 (br s, 2H, β-H), 8.89 (br s, 6H, β-H), 8.23 (dd, 8H, *J* = 19.7, 6.8 Hz, Ph), 7.92 (br s, 1H, Ph), 7.78 (dd, 10H, *J* = 13.7, 7.3 Hz, Ph), 6.38 (br s, 1H, NH), 5.75 (br s, 1H, NH), 4.70 (br s, 1H, carborane CH), 4.29 (br s, 1H, carborane CH), 4.17 (q, 2H, *J* = 7.3 Hz, CH_2_), 3.83 (br s, 3H, CH_3_), −2.71 (br s, 2H, NH). ^11^B NMR (128.3 MHz, CDCl_3_), δ (ppm): −4.7 (br s, 3B), −13.8 (br s, 7B). LC-MS (ESI): *m*/*z* calcd for ‘C_52_H_48_B_10_N_10_O_2′_ [M]^−^ 953.1; found: 952.4 [M]^−^ (negative ion mode).

5-{4-[[4-(*o*-Carborane-3′-yl)amino]-[6-((2-hydroxyethyl)thio)]-(1,3,5)-triazine-2-yl]-aminophenyl}-10,15,20-triphenylporphyrin (**19**)

To a solution of porphyrin **12** (35 mg, 0.039 mmol) in THF (5 mL) mercaptoethanol (**15**) (14 μL, 0.20 mmol) in THF (5 mL) DIPEA (43 μL, 0.25 mmol) and DMAP (15 mg, 0.12 mmol) were added and the reaction mixture was boiled for 6 h under argon untill TLC analysis revealed complete disappearance of starting porphyrin **12** (TLC control, CH_2_Cl_2_). After that, the reaction was quenched with water (150 mL) and extracted with CHCl_3_ (2×20 mL). The organic phase was washed with water and dried over Na_2_SO_4_. The solvent was removed under reduced pressure. The residue was purified by column chromatography on silica gel using CH_2_Cl_2_-EtOAc (8:2) mixture as an eluent. Yield 27 mg (74%), purple solid. UV-vis (CH_2_Cl_2_) λ_max_/nm (log *ε*): 420 (5.30), 515 (3.92), 551 (3.64), 590 (3.33), 647 (3.41). IR (KBr, cm^−1^) ν_max_: 3419 (OH), 3269 (NH), 3062 (carborane CH), 2593 (BH), 1596 (C=N). ^1^H NMR (400.1 MHz, CDCl_3_), δ (ppm): 8.87 (s, 8H, β-H), 8.23 (dd, 8H, *J* = 6.1, 4.3 Hz, Ph), 7.78 (dd, 11H, *J* = 12.0, 6.1 Hz, Ph), 5.55 (br s, 1H, NH), 4.61 (br s, 2H, carborane CH), 4.00 (br s, 2H, CH_2_), 3.36 (br s, 2H, CH_2_), −2.75 (br s, 2H, NH). ^11^B NMR (128.3 MHz, CDCl_3_), δ (ppm): −4.3 (br m, 3B), −13.6 (br m, 7B). LC-MS (ESI): *m*/*z* calcd for ‘C_51_H_47_B_10_N_9_OS’ [M]^−^ 942.2; found: 941.6 [M]^−^ (negative ion mode).

5-{4-[[4-(*o*-Carborane-3′-yl)amino]-[6-(1,1,1,3,3,3-hexafluoro-propan-2-yl)oxy]-(1,3,5)-triazine-2-yl]aminophenyl}-10,15,20-triphenylporphyrin (**20**)

To a solution of porphyrin **12** (60 mg, 0.067 mmol) in HFIP **16** (2mL) was added DIPEA (140 μL, 0.804 mmol), and this was stirred under reflux in argon for 5 h. The mixture was poured into water and extracted with chloroform. The organic layer was dried over Na_2_SO_4_, the solvent was removed under reduced pressure and the residue was purified by column chromatography on silica gel using chloroform as an eluent. Yield 49 mg (71%), purple solid. UV-vis (CH_2_Cl_2_) λ_max_/nm (log *ε*): 419 (5.49), 515 (4.24), 550 (3.96), 590 (3.81), 646 (3.74). IR (KBr, cm^−1^) ν_max_: 3318 (NH), 3063 (carborane CH), 2595 (BH), 1593 (C=N), 1280 and 1226 (CF_3_). ^1^H NMR (400.1 MHz, CDCl_3_), δ (ppm): 8.89 (s, 6H, β-H), 8.84 (br s, 2H, β-H), 8.26 (br s, 8H, Ph), 7.79 (m, 11H, Ph), 7.63 (br s, 1H, NH), 6.41 (br s, 1H, carborane NH), 5.71 (br s, 1H, CH), 4.61 (br s, 1H, carborane CH), 4.51 (br s, 1H, carborane CH), −2.73 (br s, 2H, NH). ^11^B NMR (128.3 MHz, CDCl_3_), δ (ppm): −4.6 (br s, 3B), −13.8 (br s, 7B). ^19^F NMR (376.5 MHz, CDCl_3_,), δ ppm: −72.8 (s, 2F), −73.0 (s, 4F). LC-MS (ESI-MS): *m*/*z* calcd for ‘C_52_H_43_B_10_F_6_N_9_O’ [M]^−^ 1032.1; found: 1031.4 [M]^−^ (negative ion mode).

5-{4-[[4-(*m*-Carborane-9′-yl)thio]-[6-(aminoethylthio)]-(1,3,5)-triazine-2-yl]amino-phenyl}-10,15,20-triphenylporphyrin (**21**)

To a solution of porphyrin **3** prepared from cyanuric chloride **2** (12 mg, 0.065 mmol), DIPEA (13 μL, 0.075 mmol) and porphyrin **1** (40 mg, 0.064 mmol) in THF (6 mL) in situ as described above for the compound **5** the THF solution (3 mL) 9-mercapto-*m*-carborane (**4**) (26 mg, 0.148 mmol) and DIPEA (31 μL, 0.177 mmol) were added, and the mixture was stirred at room temperature for 8 h. An excess 2-aminoethanethiol hydrochloride (**23**) (52 mg, 0.458 mmol) and DIPEA (96 μL, 0.551 mmol) were added, and the mixture was stirred under reflux for 15 h. After the reaction was completed (TLC control, hexane -CHCl_3_, 2:3), resulting mass was treated with water (100 mL), extracted with CH_2_Cl_2_ (2×20 mL), organic phase was dried over Na_2_SO_4_. After removal of the solvent in vacuo, the residue was purified by column chromatography on silica gel using CH_2_Cl_2_-hexane system (1:1) as an eluent. Yield 54 mg (85%), purple solid. UV-vis (CH_2_Cl_2_) λ_max_/nm (log *ε*): 420 (5.05), 518 (3.70), 552 (3.48), 592 (3.25), 647 (3.20). IR (KBr, cm^−1^) ν_max_: 3317 (NH), 3054 (carborane CH), 2602 (BH), 1570 (C=N). ^1^H NMR (400.1 MHz, CDCl_3_), δ (ppm): 8.90 (m, 8H, β-H), 8.24 (br s, 8H, Ph), 8.02 (br s, 2H, Ph), 7.77 (br s, 9H, Ph), 3.90 (br s, 2H, CH_2_), 3.74 (br s, 2H, CH_2_), 2.98 (br s, 2H, NH_2_), 2.90 (br s, 2H, carborane CH), −2.72 (br s, 2H, NH). ^11^B NMR (128.3 MHz, CDCl_3_), δ (ppm): 0.7 (br s, 1B, B^9^), −6.4 (d, 2B, *J* = 151 Hz), −9.8 (d, 1B, *J* = 156 Hz), −13.9 (d, 4B, *J* = 154 Hz), −17.5 (d, 2B, *J* = 185 Hz). MS (MALDI-TOF): *m*/*z* calcd for ‘C_51_H_47_B_10_N_9_S_2_*HCl’ [M]^+^ 994.68; found: 994.38 [M]^+^.

5-{4-[[4,6-Bis((4-aminophenyl)amino)]-(1,3,5)-triazine-2-yl]aminophenyl}-10,15,20-triphenylporphyrin (**26**)

To a solution of porphyrin **3** prepared from cyanuric chloride **2** (30 mg, 0.163 mmol), DIPEA (32 μL, 0.184 mmol) and porphyrin **1** (103 mg, 0.163 mmol) in THF (10 mL) in situ as described above for the compound **5** the solution of 1,4-phenylenediamine **27** (85 mg, 0.787 mmol), DIPEA (170 μL, 0.976 mmol) and DMAP (10 mg, 0.082 mmol) in THF (4 mL) were added, and the mixture was boiled at 65 °C for 30 h until the reaction completion. The mixture was treated with water (100 mL), extracted with CH_2_Cl_2_ (3×20 mL), and the organic solution was dried over Na_2_SO_4_. After removal of the solvent in vacuo, the residue was purified by column chromatography on silica gel using CH_2_Cl_2_-acetone (8:2) as an eluent to give conjugate **26** (95 mg, yield 64%) as a purple solid. UV-vis (THF) λ_max_/nm (log *ε*): 419 (5.22), 516 (3.96), 551 (3.75), 592 (3.49), 649 (3.44). IR (KBr, cm^−1^) ν_max_: 3397 (NH), 3319 (NH_2_), 1568 (C=N). ^1^H NMR (400.1 MHz, CDCl_3_), δ (ppm): 8.99 (s, 1H, NH), 8.98 (s, 1H, NH), 8.91 (s, 8H, β-H), 8.25 (dd, 8H, *J* = 10.2, 6.9 Hz, Ph), 8.15 (d, 2H, *J* = 7.3 Hz, Ph), 7.97 (d, 3H, *J* = 7.9 Hz, Ph), 7.76 (m, 10H, Ph), 7.36 (d, 4H, *J* = 7.9 Hz, Ph), 3.37 (br s, 4H, NH_2_), −2.68 (br s, 2H, NH). LC-MS (ESI-MS): *m*/*z* calcd for ‘C_59_H_44_N_12′_ [M]^+^ 921.1; found: 921.5 [M]^+^ (positive ion mode).

Synthesis of maleimide-substituted conjugates **24**, **25**

To a solution of porphyrin **26** (70 mg, 0.076 mmol) in THF (5 mL) the solution of 3-bromo-1-(*N*-(*o*-carborane-3′-yl))maleimide (**28**) (50 mg, 0.157 mmol) and NaOAc (15 mg, 0.183 mmol) in dry MeOH (5 mL) and the reaction mixture were boiled at 65 °C for 70 h until the reaction completion. Then reaction mixture was poured into a water (50 mL), extracted with EtOAc (3×20 mL), the combined organic solution was dried over Na_2_SO_4_, filtered and the solvents were evaporated in vacuo. After removal of the solvent in vacuo, the residue was purified by column chromatography on silica gel using chloroform-acetone system (7:3) as an eluent. Yield of *nido*- maleimide-substituted conjugate **24** 89%, *closo*- maleimide-substituted conjugate **25** 8%.

5-{4-[[4,6-Bis((4-(1-(*N*-(7,8-dicarbaundecaborate-3′-yl))maleimido)aminophenyl)-amino]-(1,3,5)-triazine-2-yl]aminophenyl}-10,15,20-triphenylporphyrin (**24**)

Yield 93 mg (89%), green solid. UV-vis ((CH_3_)_2_CO) λ_max_/nm (log *ε*): 416 (5.38), 514 (4.18), 549 (3.96), 591 (3.70), 647 (3.70). IR (KBr, cm^−1^) ν_max_: 3405 (NH), 3055 (carborane CH), 2521 (BH), 1692 (C=O), 1643 (C=C), 1576 (C=N). ^1^H NMR (400.1 MHz, (CD_3_)_2_CO), δ (ppm): 8.96 (br s, 2H, NH), 8.80 (br s, 8H, β-H), 8.24 (br s, 2H, Ph), 8.15 (br s, 8H, Ph), 7.89 (br s, 3H, Ph), 7.68 (t, 10H, *J* = 5.4 Hz, Ph), 7.34 (d, 4H, *J* = 6.9 Hz, Ph), 5.45 (br s, 2H, CH=C), 2.55 (br s, 4H, carborane CH), −2.71 (br s, 2H, NH). ^11^B NMR (128.3 MHz, (CD_3_)_2_CO), δ (ppm): −11.4 (br s, 6B, (B^3^ and 2B)), −17.4 (d, 4B, *J* = 130 Hz), −21.7 (d, 4B, *J* = 135 Hz), −38.1 (d, 4B, *J* = 133 Hz). LC-MS (ESI-MS): *m*/*z* calcd for ‘C_71_H_66_B_18_N_14_O_4_’ [M]^2−^ 686.9; found: 687.0 [M]^2−^ (negative ion mode).

5-{4-[[4,6-Bis((4-(1-(*N*-(*o*-carborane-3′-yl))maleimido)aminophenyl)amino]-(1,3,5)-triazine-2-yl]aminophenyl}-10,15,20-triphenylporphyrin (**25**)

Yield 9.0 mg (8%), purple solid. UV-vis (CHCl_3_) λ_max_/nm (log *ε*): 421 (5.26), 515 (3.93), 554 (3.72), 594 (3.59), 651 (3.81). IR (KBr, cm^−1^) ν_max_: 3320 (NH), 3074 (carborane CH), 2596 (BH), 1765, 1704 (C=O), 1640 (C=C), 1576 (C=N). ^1^H NMR (400.1 MHz, (CD_3_)_2_CO), δ (ppm): 8.86 (br s, 8H, β-H), 8.31 (d, 2H, *J* = 6.4 Hz, Ph), 8.23 (d, 4H, *J* = 5.1 Hz, Ph), 8.17 (d, 4H, *J* = 8.3 Hz, Ph), 7.94 (m, 3H, Ph), 7.82 (t, 10H, *J* = 5.9 Hz, Ph), 7.42 (d, 4H, *J* = 8.6 Hz, Ph), 5.60 (br s, 2H, CH=C), 5.00 (br s, 4H, carborane CH), −2.73 (br s, 2H, NH). ^11^B NMR (128.3 MHz, (CD_3_)_2_CO), δ (ppm): −4.4 (d, 4B, *J* = 147 Hz), −8.0 (br s, 2B, B^3^), −10.0 (d, 2B, *J* = 163 Hz), −13.2 (d, 12B, *J* = 168 Hz). LC-MS (ESI): *m*/*z* calcd for ‘C_71_H_64_B_20_N_14_O_4_’ [M-2H]^−^ 1393.6; found: 1393.2 [M-2H]^−^ (negative ion mode).

### 3.3. Fluorescence Spectroscopy

For compounds **8**, **9**, **17**, **18** and **24** fluorescence spectra (after excitation 405 nm) were measured in ethanol in quartz cuvettes (10 mm × 10 mm) on a Shimadzu UV-3101 PC spectrophotometer (Shimadzu, Japan) and on a Panorama fluorometer (Russia).

### 3.4. Cell Culture

The human colon adenocarcinoma cell line HCT116 was obtained from American Type Culture Collection (Manassas, VA, USA). Cells were propagated in Dulbecco’s modified Eagle’s medium supplemented with 10% fetal bovine serum (HyClone, Logan, UT, USA), 2 mM L-glutamine, 100 U/mL penicillin and 100 µg/mL streptomycin (PanEco, Russia) at 37 °C, 5% CO_2_ in a humidified atmosphere. Cells in logarithmic phase of growth were used in the experiments.

### 3.5. Cytotoxicity Studies

The cytotoxicity of compounds for HCT116 human colon carcinoma cells was assessed in MTT assays. Cells were plated in 96 well plates (NUNC, USA, 5·10^3^ cells in 190 µl culture medium per well). After 24 h at 37 °C, 5% CO_2_ in a humidified atmosphere, cells were treated with compounds dissolved from 10 mM stocks in DMSO. Dark cytotoxicity was assessed in a formazan conversion assay (MTT-test) after a 72 h exposure. For photoinduced cytotoxicity, cells were loaded with tested compounds for 24 h, the medium was replaced with a fresh one and cells were illuminated with a 400 nm laser, 2 J/cm^2^ (AFC Polironics, Russia). After the completion of drug exposure in the dark or drug accumulation/illumination, 0.5 mg/ml 3-(4,5-dimethylthiazol-2-yl)-2,5-diphenyltetrazolium bromide (MTT reagent) was added to cells for 2 h, the culture medium was removed, cells were resuspended in 100 µL DMSO and the optical densities were measured on a Multiscan FC plate spectrophotometer (Thermo Scientific, Waltham, MA, USA) at a wavelength of 571 nm. The percentage of survived cells for each dose was calculated as the quotient of the average optical density in the wells after incubation with this dose to the average optical density of the control wells (the values of the latter are taken as 100%). Four independent experiments were performed for each concentration. Standard deviations did not exceed 10%.

### 3.6. Confocal Laser Scanning Fluorescence Microscopy

HCT116 cells were grown in 35 mm Petri dishes with a glass bottom (SPL, Korea) until 50% confluence. Then, tested compounds (10 µM each) were added for an additional 24 h at 37 °C, 5% CO_2_. After the completion of incubations, cells were washed with PBS and stained with the following probes according to manufacturer’s recommendations (all from Invitrogen Corp., Carlsbad, CA, USA): LysoTracker™ Green DND-26 (1 µM, 30 min), DHR 123 (1 µM, 20 min), the nuclear dye Hoechst 33342 (0.1 µg/ml, 10 min). After the completion of staining, cells were washed with PBS and analyzed by confocal microscopy at the following excitation and emission wavelengths: LysoTracker™ Green (λ_ex_ = 476 nm/λ_em_ = 490–560 nm), DHR 123 (λ_ex_ = 476 nm/λ_em_ = 490–560 nm), Hoechst 33342 (λ_ex_ = 405 nm/λ_em_ = 415–470 nm), compounds **9**, **24** (λ_ex_ = 405 nm/λ_em_ = 600–750 nm). To detect necrotic cells, PI (10 µg/ml) was added to the cells and analyzed at λ_ex_ = 488 nm/λ_em_ = 610–730 nm. Laser scanning confocal microscope Leica TCS SPE 5 with LAS AF software (Leica Microsystems GmbH, Wetzlar, Germany) was used for registration.

## 4. Conclusions

A convenient synthetic approach for the preparation of *s*-triazines containing porphyrin and carborane clusters was developed by nucleophilic substitution of the chlorine atom in cyanuric chloride with 5-(4-aminophenyl)-10,15,20-triphenylporphyrin, followed by the substitution of remaining chlorine atoms with carborane clusters or other *S*, *N*, *O*-nucleophile functionalities. The prepared compounds were fully characterized by UV-vis, IR, ^1^H and ^11^B NMR spectroscopy and mass-spectrometry. As a result, it was shown that cyanuric chloride is a convenient scaffold for the creation of multicomponent systems efficient for biomedical and other application.

## Data Availability

Not applicable.

## Supplementary Materials

The following supporting information can be downloaded at: https://www.mdpi.com/article/10.3390/molecules27196200/s1, Figure S1. The ^1^H, ^11^B NMR spectra of **5 in CDCl_3_;** Figure S2. The ^1^H, ^11^B NMR spectra of **6 in (CD_3_)_2_CO;** Figure S3. The ^1^H and ^11^B NMR spectra of **8 in CDCl_3_;** Figure S4. The ^1^H, ^11^B NMR spectra of **9 in CDCl_3_;** Figure S5. The ^1^H and ^11^B NMR spectra of **11 in CDCl_3_;** Figure S6. The ^1^H and ^11^B NMR spectra of **12 in CDCl_3_;** Figure S7. The ^1^H and ^11^B NMR spectra of **17 in CDCl_3_;** Figure S8. The ^1^H and ^11^B NMR spectra of **18 in CDCl_3_;** Figure S9. The ^1^H and ^11^B NMR spectra of **19 in CDCl_3_;** Figure S10. The ^1^H, ^11^B and ^19^F spectra of **20 in CDCl_3_;** Figure S11. The ^1^H, ^11^B NMR spectra of **21 in CDCl_3_;** Figure S12. The ^1^H and ^11^B NMR spectra of **24 in (CD_3_)_2_CO;** Figure S13. The ^1^H and ^11^B NMR spectra of **25 in (CD_3_)_2_CO;** Figure S14. Dark cytotoxicity of compounds **8**, **9**, **17**, **18** and **24**; Figure S15. Photoinduced cytotoxicity of compounds **8**, **9**, **17**, **18** and **24**.

## Sample Availability

Samples of the compounds are available from the authors.
