# Supplementary material for: Multicomponent Molecular Systems Based on Porphyrins, 1,3,5-Triazine and Carboranes: Synthesis and Characterization
Source: Molecules. 2022 Sep 21;27(19):6200. doi: 10.3390/molecules27196200 (PMC9572311; doi:10.3390/molecules27196200)
Supplement: Supplementary file 1 [file molecules-27-06200-s001.zip › molecules-1928035-supplementary.pdf]

Multicomponent Molecular Systems Based on Porphyrins, 1,3,5-Triazine and Carboranes: Synthesis and Characterization

Victoria M. Alpatova [1], Evgeny G. Rys [1], Elena G. Kononova [1], Ekaterina A. Khakina [1], Alina A. Markova [1,2], Anna V. Shibaeva [2], Vladimir A. Kuzmin [2] and Valentina A. Ol'shevskaya [1,*]

[1] A.N.Nesmeyanov Institute of Organoelement Compounds, Russian Academy of Sciences, 28, bld. 1 Vavilova St., 119334 Moscow, Russian Federation
[2] Emanuel Institute of Biochemical Physics, 4 Kosygina st., Russian Academy of Sciences, 119334 Moscow, Russian Federation
* Correspondence: olshevsk@ineos.ac.ru

Table of Contents

1. The ^{1}H and ^{11}B NMR spectra of 5-{4-[[4,6-bis(*m*-carborane-9′-yl)thio]-(1,3,5)-triazine-2-yl]aminophenyl}-10,15,20-triphenylporphyrin (**5**) 3
2. The ^{1}H and ^{11}B NMR spectra 5-{4-[[4,6-bis(*m*-carborane-9′-yl)thio]-(1,3,5)-triazine-2-yl]aminophenyl}-10,15,20-triphenylporphyrinato zinc (II) (**6**) 3
3. The ^{1}H and ^{11}B NMR spectra of 5-{4-[[4,6-bis(*o*-carborane-1′-yl)thio]-(1,3,5)-triazine-2-yl]aminophenyl}-10,15,20-triphenylporphyrin (**8**) 4
4. The ^{1}H and ^{11}B NMR spectra of 5-{4-[[4,6-bis(*o*-carborane-1′-yl)thio]-(1,3,5)-triazine-2-yl]aminophenyl}-10,15,20-triphenylporphyrinato zinc (II) (**9**) 5
5. The ^{1}H and ^{11}B NMR spectra of 2-[(*o*-carborane-3′-yl)amino]-4,6-dichloro-(1,3,5)-triazine (**11**) 5
6. The ^{1}H and ^{11}B NMR spectra of 5-{4-[[4-(*o*-carborane-3′-yl)amino]-6-chloro-(1,3,5)-triazine-2-yl]aminophenyl}-10,15,20-triphenylporphyrin (**12**) 6
7. The ^{1}H and ^{11}B NMR spectra of 5-{4-[[4-(*o*-carborane-3′-yl)amino]-(6-carboxymethylthio)-(1,3,5)-triazine-2-yl]-aminophenyl}-10,15,20-triphenylporphyrin (**17**) 7

8. The ^{1}H and ^{11}B NMR spectra of 5-{4-[[4-(*o*-carborane-3′-yl)amino]-(6-methoxycarbonylmethylamino)-(1,3,5)-triazine-2-yl]aminophenyl}-10,15,20-triphenylporphyrin (**18**) 7

9. The ^{1}H and ^{11}B NMR spectra of 5-{4-[[4-(*o*-carborane-3′-yl)amino]-[6-((2-hydroxyethyl)thio)]-(1,3,5)-triazine-2-yl]-aminophenyl}-10,15,20-triphenylporphyrin (**19**) 8

10. The ^{1}H, ^{11}B and ^{19}F NMR spectra of 5-{4-[[4-(*o*-carborane-3′-yl)amino]-[6-(1,1,1,3,3,3-hexafluoro-propan-2-yl)oxy]-(1,3,5)-triazine-2-yl]aminophenyl}-10,15,20-triphenylporphyrin (**20**) 9

11. The ^{1}H and ^{11}B NMR spectra of 5-{4-[[4-(*m*-carborane-9′-yl)thio]-[6-(aminoethylthio)]-(1,3,5)-triazine-2-yl]aminophenyl}-10,15,20-triphenylporphyrin (**21**) 10

12. The ^{1}H and ^{11}B NMR spectra of 5-{4-[[4,6-bis((4-(1-(*N*-(7,8-dicarbaundecaborate-3′-yl))maleimido)aminophenyl)-amino]-(1,3,5)-triazine-2-yl]aminophenyl}-10,15,20-triphenylporphyrin (**24**) 10

13. The ^{1}H and ^{11}B NMR spectra of 5-{4-[[4,6-bis((4-(1-(*N*-(*o*-carborane-3′-yl))maleimido)aminophenyl)amino]-(1,3,5)-triazine-2-yl]aminophenyl}-10,15,20-triphenylporphyrin (**25**) 11

14. The dark cytotoxicity of carboranylporphyrins **8, 9, 17, 18** and **24** 12

15. The photoinduced cytotoxicity of carboranylporphyrins **8, 9, 17, 18** and **24** 12

1. The ^{1}H and ^{11}B NMR spectra of 5-{4-[[4,6-bis(*m*-carborane-9′-yl)thio]-(1,3,5)-triazine-2-yl]aminophenyl}-10,15,20-triphenylporphyrin (5)

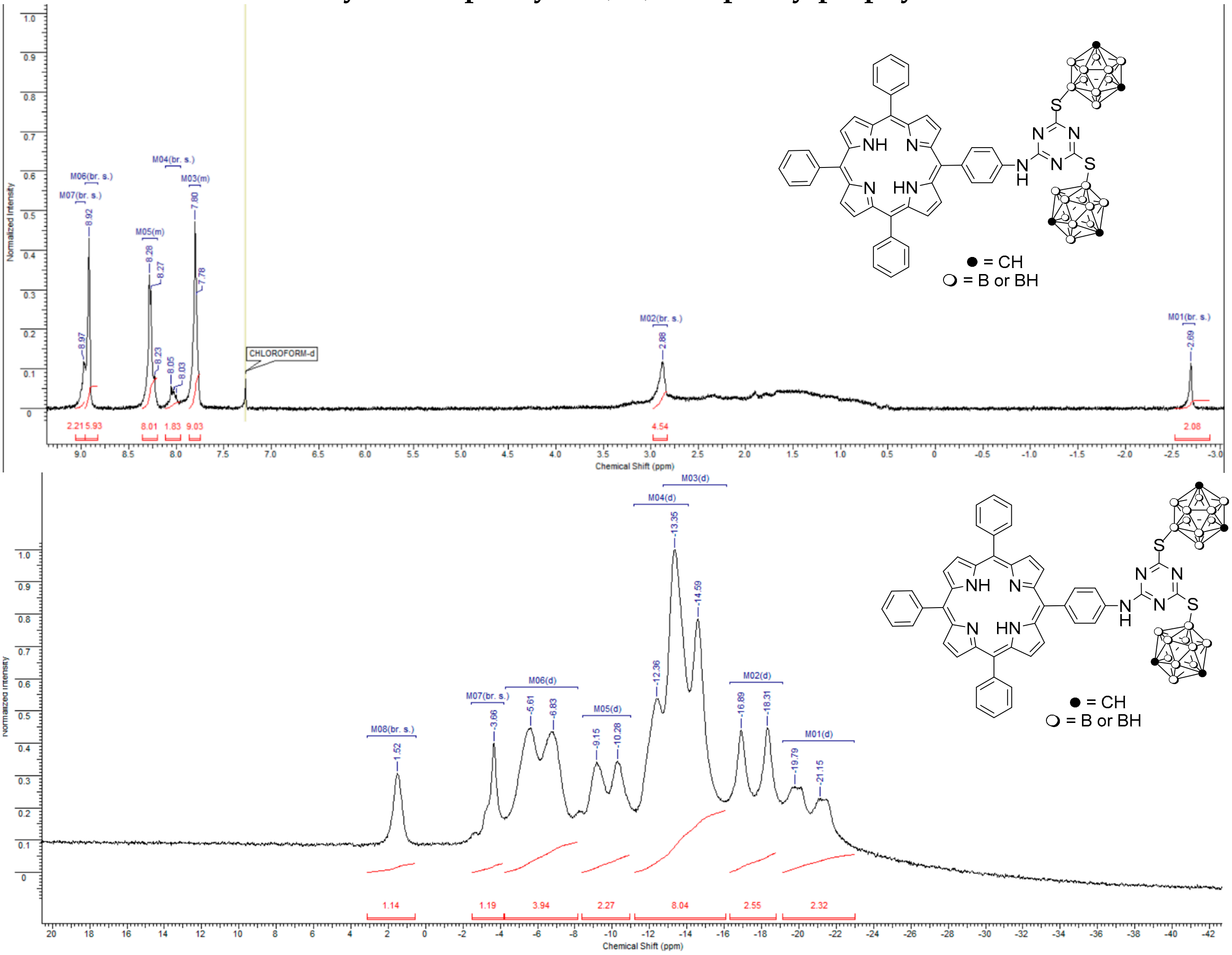

Figure S1. The ^{1}H, ^{11}B NMR spectra of **5** in $CDCl_3$.

2. The ^{1}H and ^{11}B NMR spectra 5-{4-[[4,6-bis(*m*-carborane-9′-yl)thio]-(1,3,5)-triazine-2-yl]aminophenyl}-10,15,20-triphenylporphyrinato zinc (II) (6)

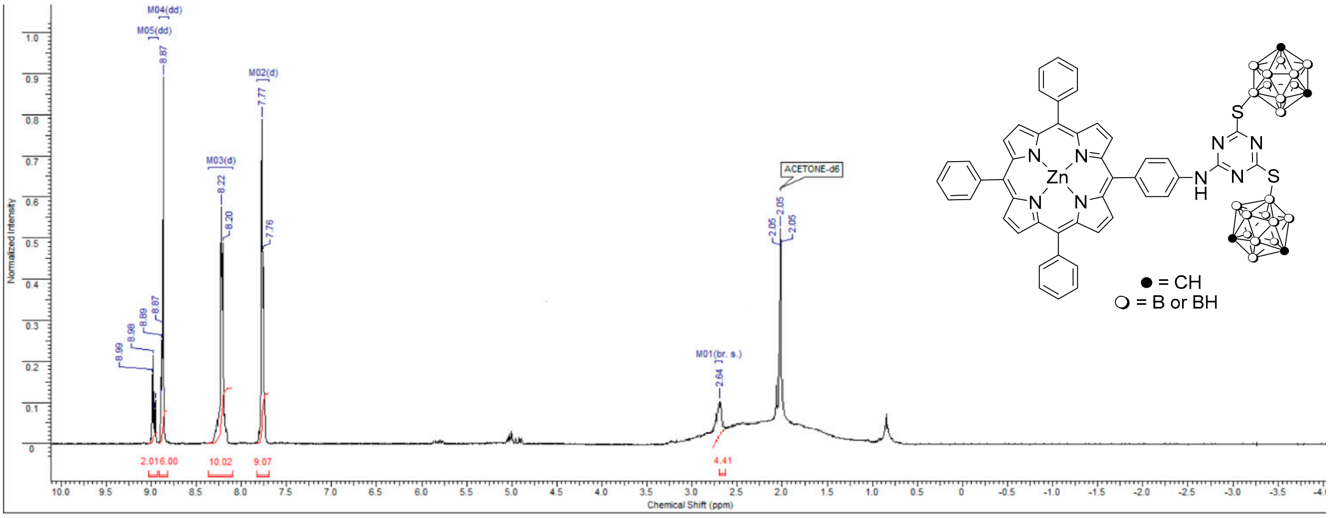

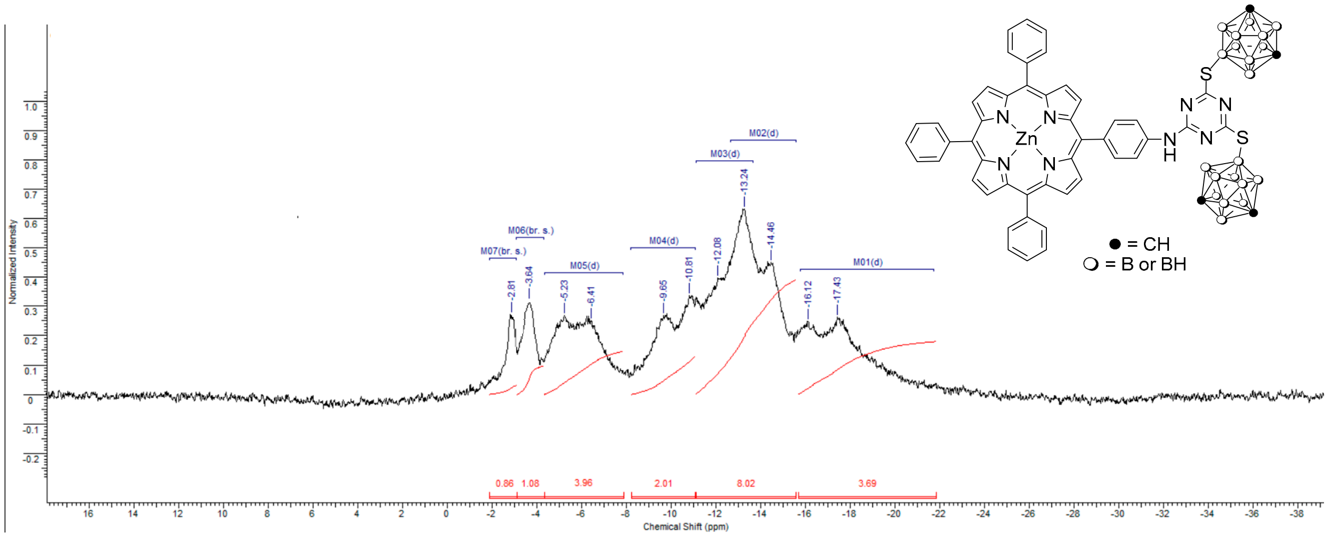

Figure S2. The ^{1}H, ^{11}B NMR spectra of **6** in $(CD_3)_2CO$.

3. The ^{1}H and ^{11}B NMR spectra of 5-{4-[[4,6-bis(*o*-carborane-1′-yl)thio]-(1,3,5)-triazine-2-yl]aminophenyl}-10,15,20-triphenylporphyrin (8)

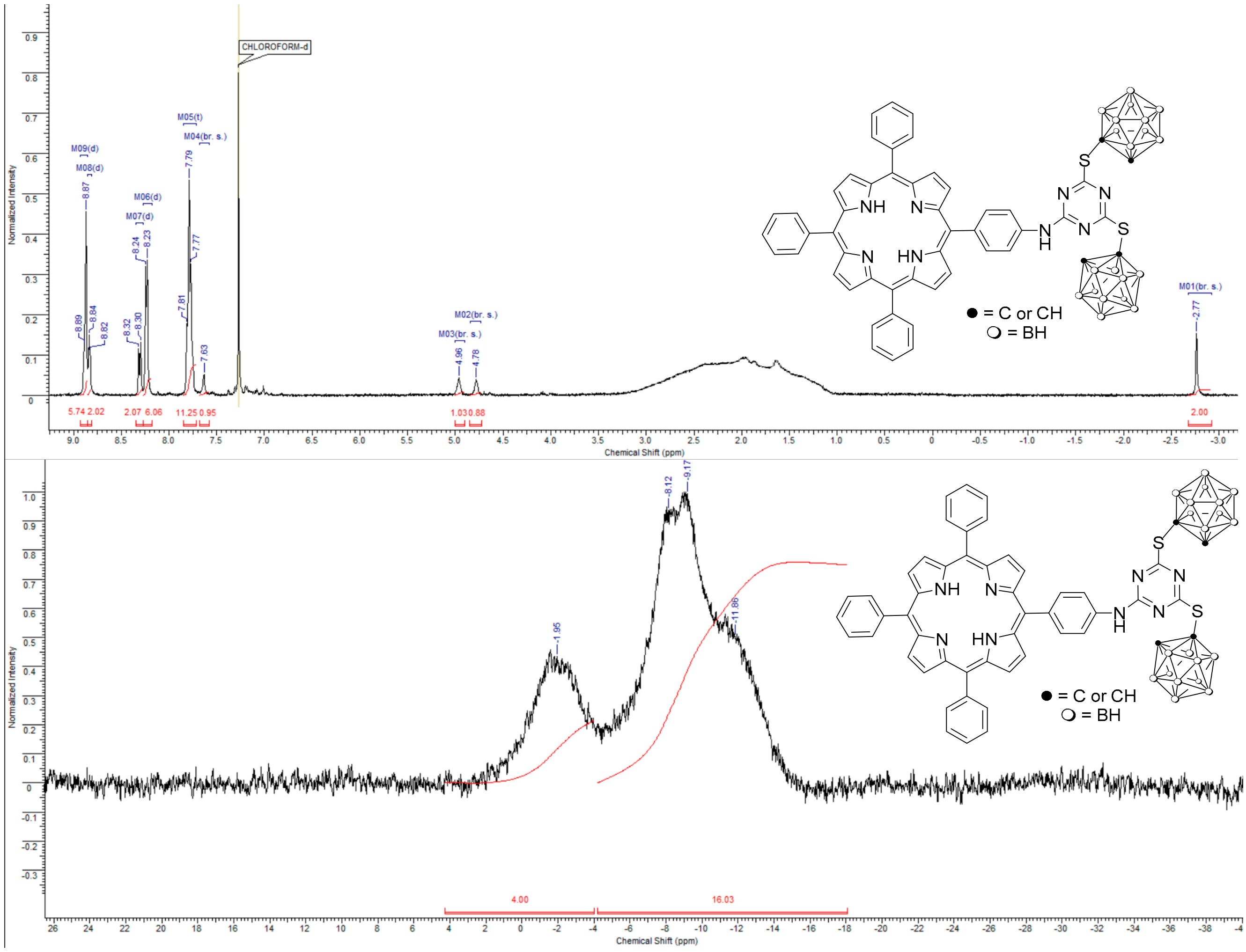

Figure S3. The ^{1}H and ^{11}B NMR spectra of **8** in $CDCl_3$.

4. The ^{1}H and ^{11}B NMR spectra of 5-{4-[[4,6-bis(*o*-carborane-1′-yl)thio]-(1,3,5)-triazine-2-yl]aminophenyl}-10,15,20-triphenylporphyrinato zinc (II) (9)

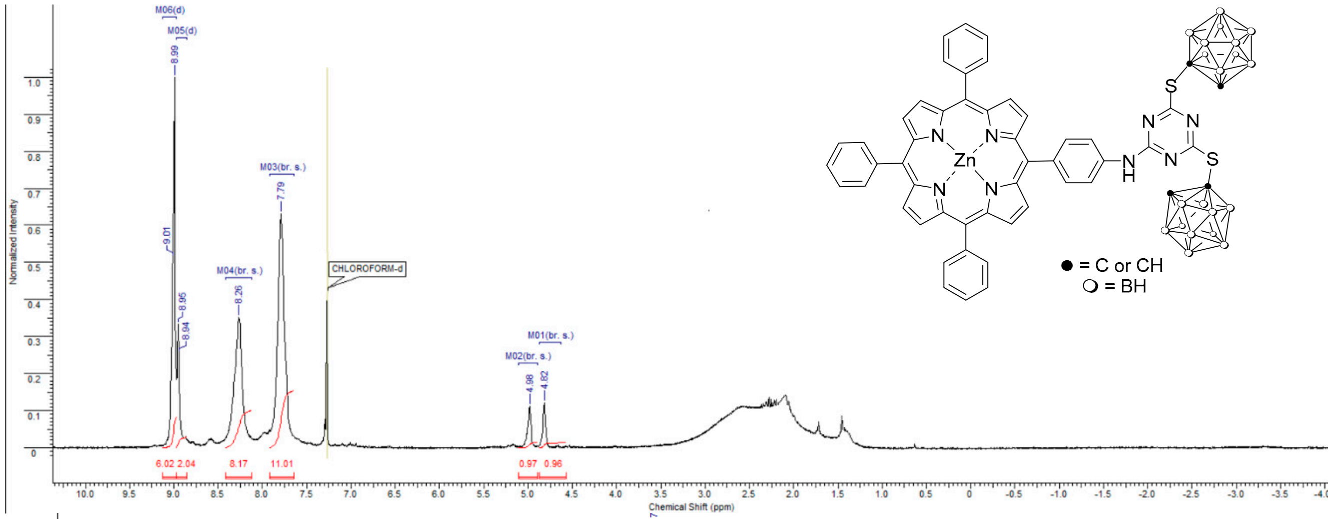

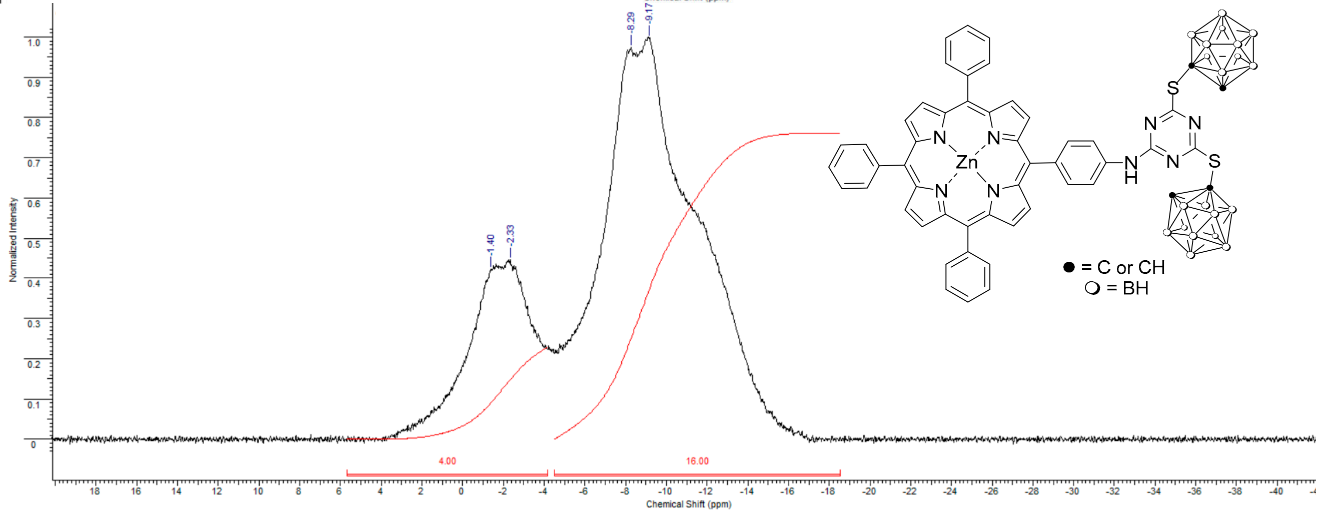

Figure S4. The ^{1}H, ^{11}B NMR spectra of **9** in $CDCl_3$.

5. The ^{1}H and ^{11}B NMR spectra of 2-[(*o*-carborane-3′-yl)amino]-4,6-dichloro-(1,3,5)-triazine (11)

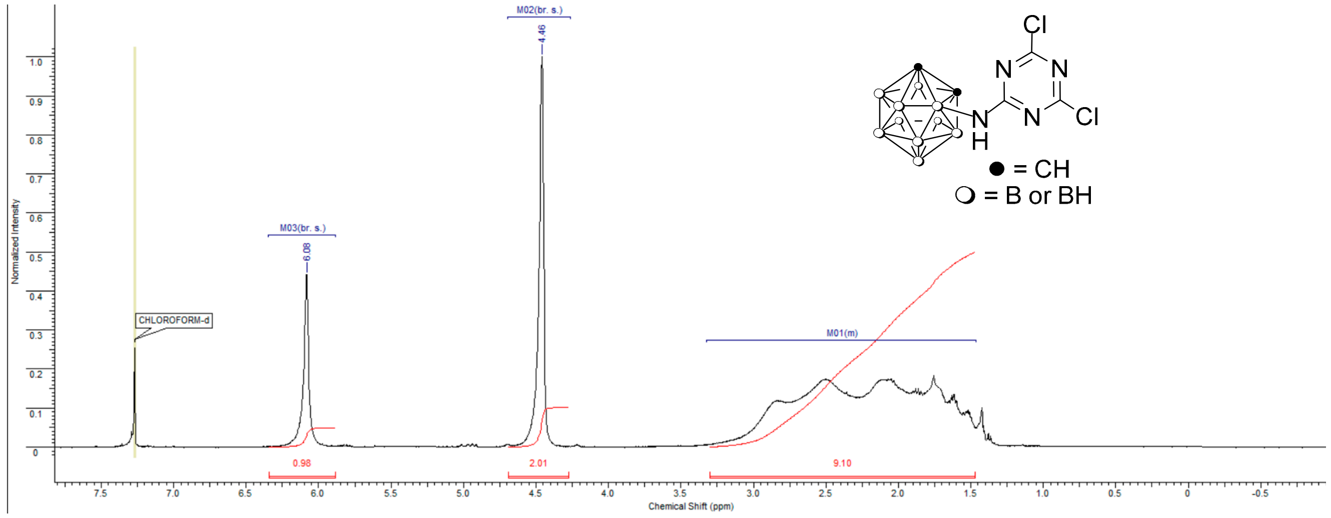

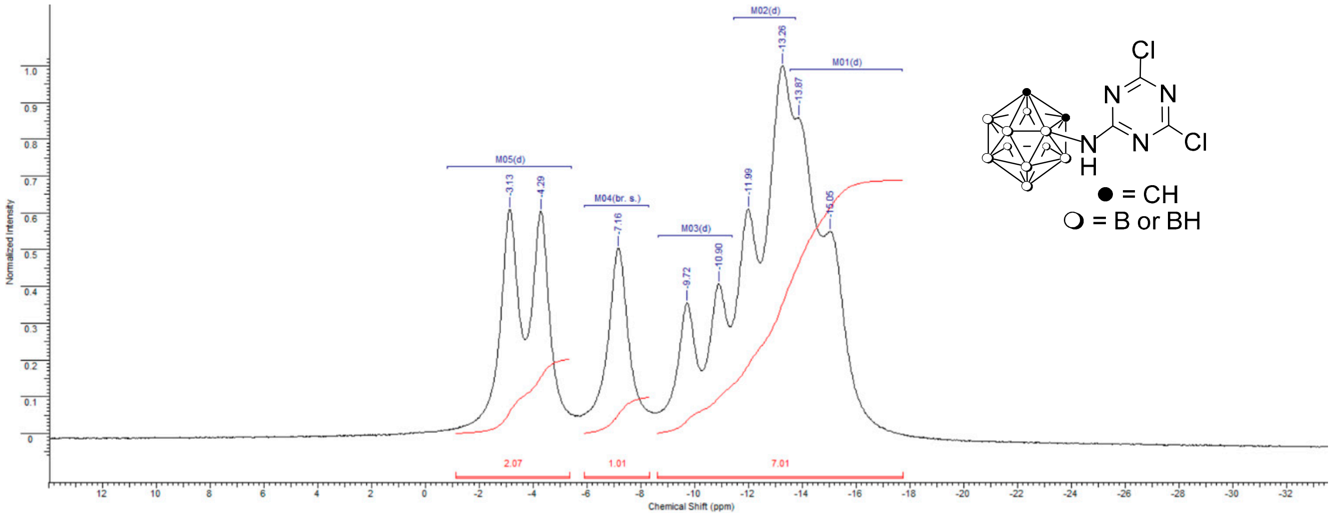

Figure S5. The ^{1}H and ^{11}B NMR spectra of **11** in $CDCl_3$.

6. The ^{1}H and ^{11}B NMR spectra of 5-{4-[[4-(*o*-carborane-3′-yl)amino]-6-chloro-(1,3,5)-triazine-2-yl]aminophenyl}-10,15,20-triphenylporphyrin (12)

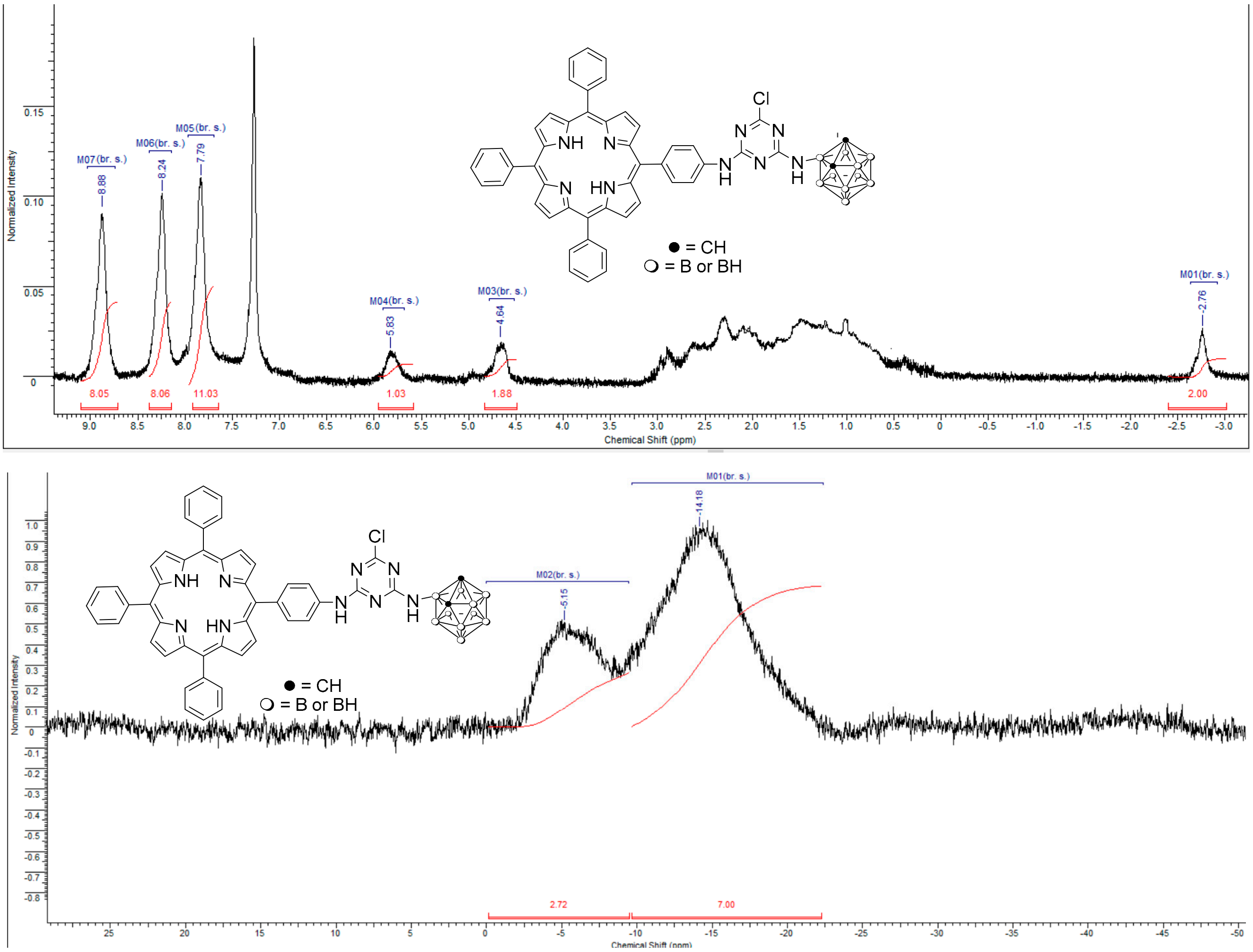

Figure S6. The ^{1}H and ^{11}B NMR spectra of **12** in $CDCl_3$.

7. The ^1H and ^{11}B NMR spectra of 5-{4-[[4-(*o*-carborane-3′-yl)amino]-(6-carboxymethylthio)-(1,3,5)-triazine-2-yl]-aminophenyl}-10,15,20-triphenylporphyrin (17)

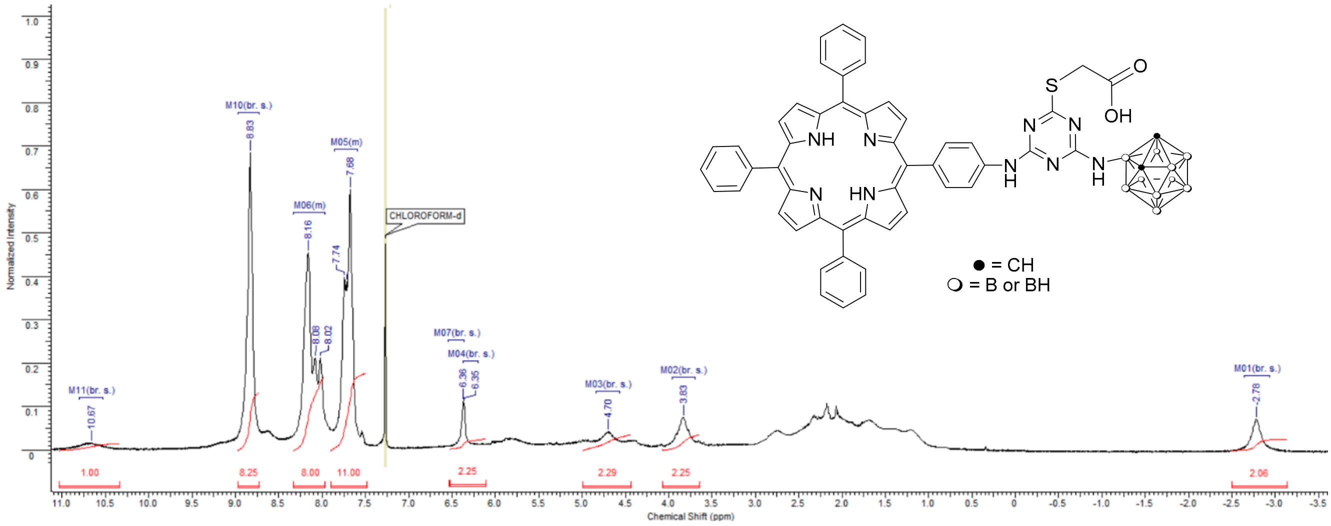

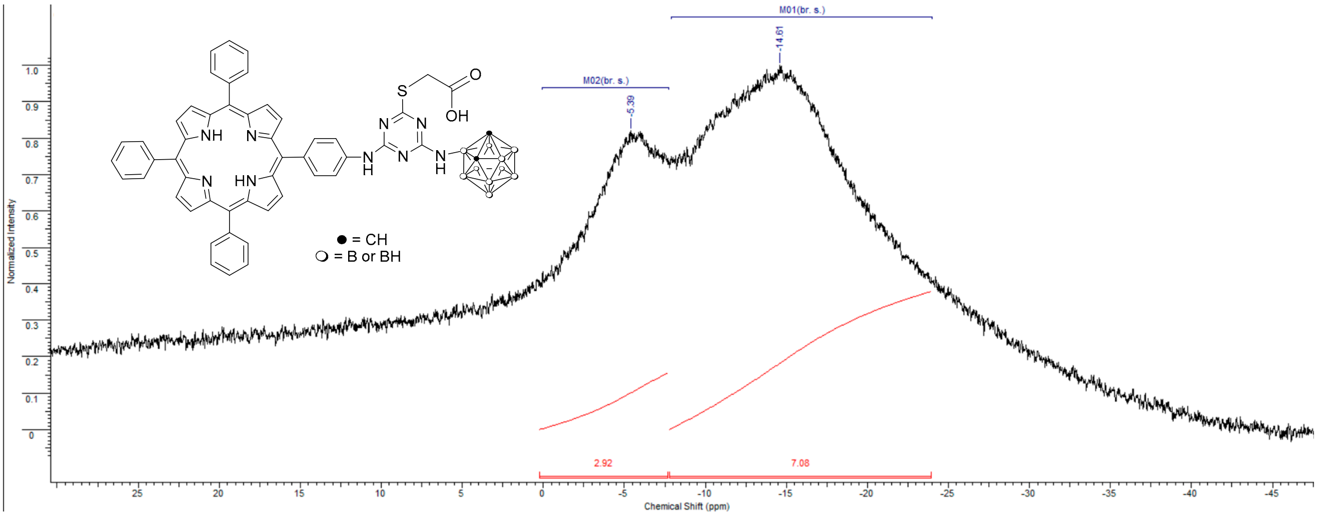

Figure S7. The ^1H and ^{11}B NMR spectra of **17** in $CDCl_3$.

8. The ^1H and ^{11}B NMR spectra of 5-{4-[[4-(*o*-carborane-3′-yl)amino]-(6-methoxycarbonylmethylamino)-(1,3,5)-triazine-2-yl]aminophenyl}-10,15,20-triphenylporphyrin (18)

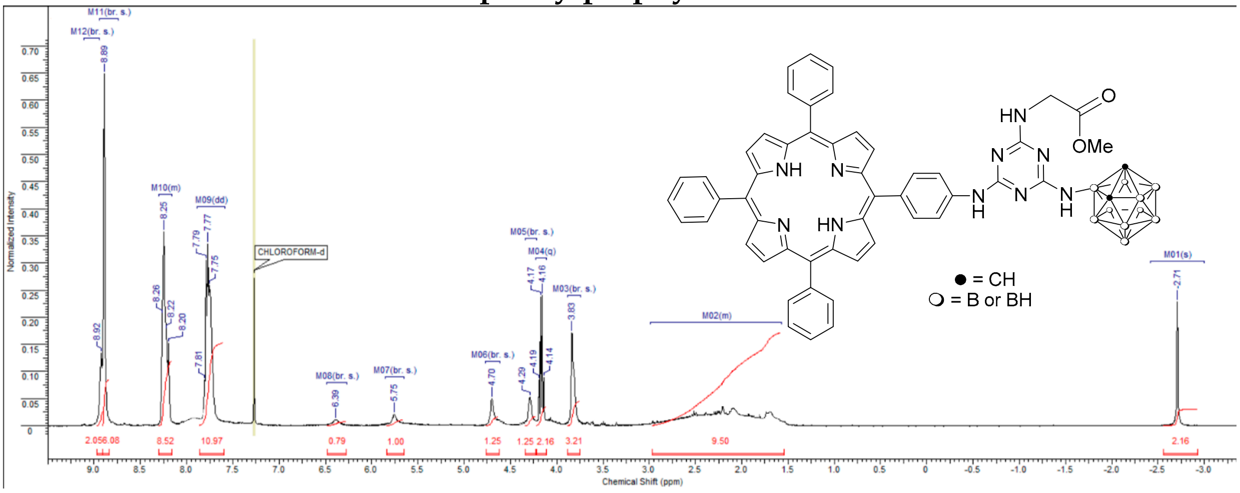

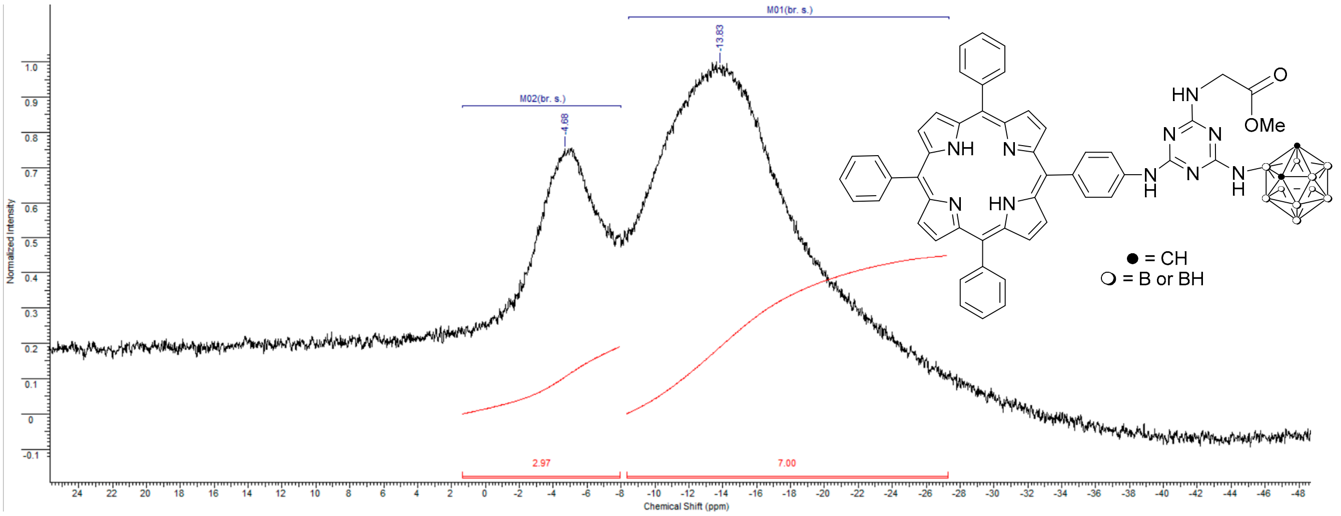

Figure S8. The ^{1}H and ^{11}B NMR spectra of **18** in $CDCl_3$.

9. The ^{1}H and ^{11}B NMR spectra of 5-{4-[[4-(*o*-carborane-3′-yl)amino]-[6-((2-hydroxyethyl)thio)]-(1,3,5)-triazine-2-yl]-aminophenyl}-10,15,20-triphenylporphyrin (19)

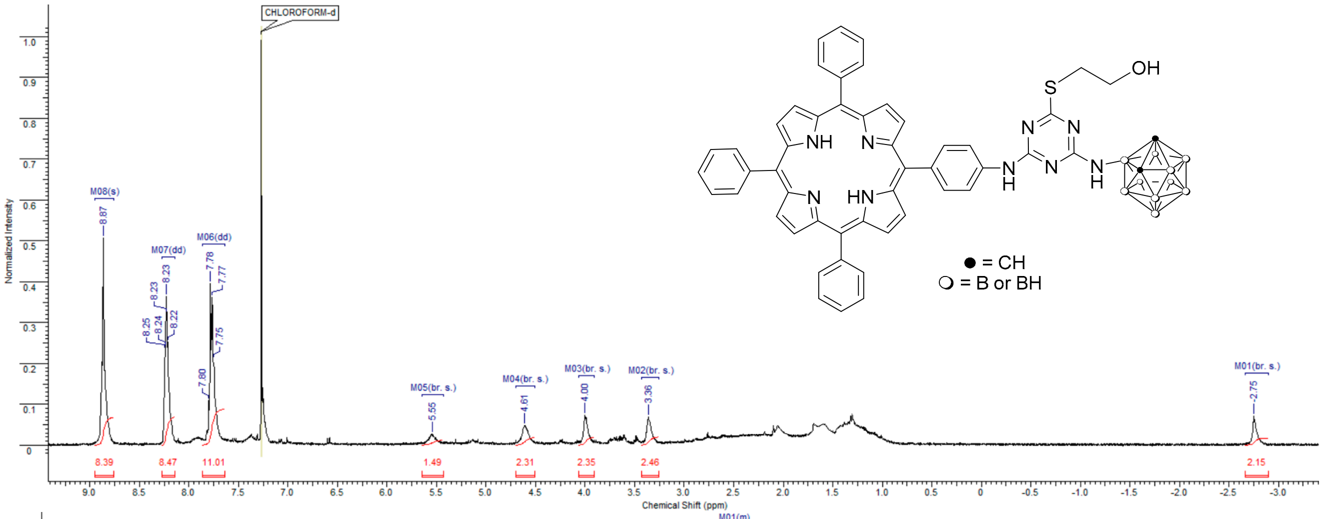

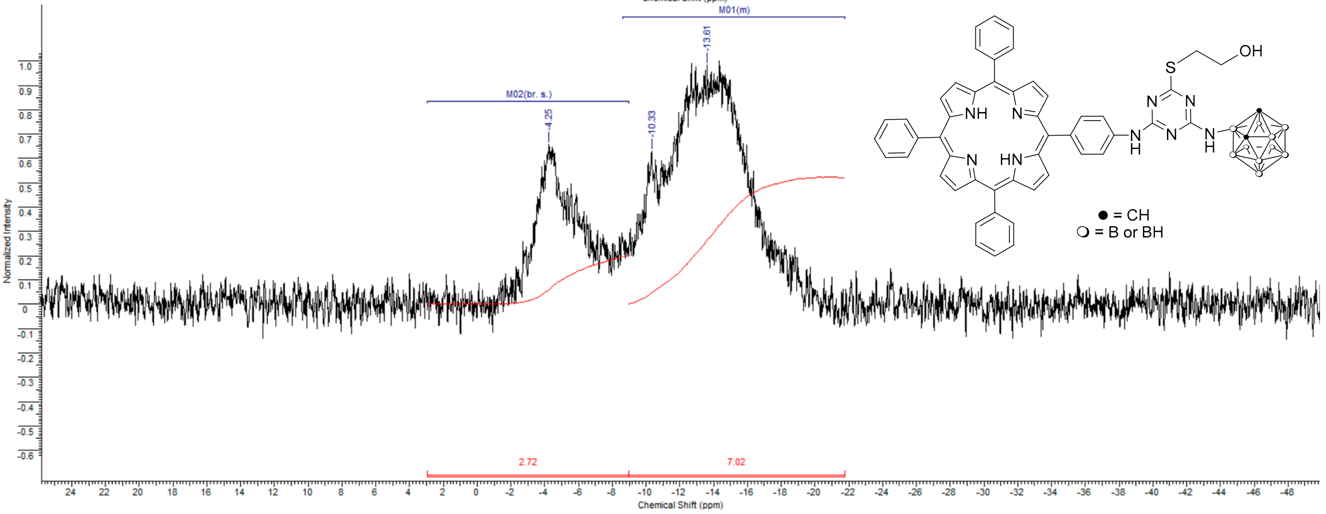

Figure S9. The ^{1}H and ^{11}B NMR spectra of **19** in $CDCl_3$.

10. The ^{1}H, ^{11}B and ^{19}F NMR spectra of 5-{4-[[4-(*o*-carborane-3′-yl)amino]-[6-(1,1,1,3,3,3-hexafluoro-propan-2-yl)oxy]-(1,3,5)-triazine-2-yl]aminophenyl}-10,15,20-triphenylporphyrin (20)

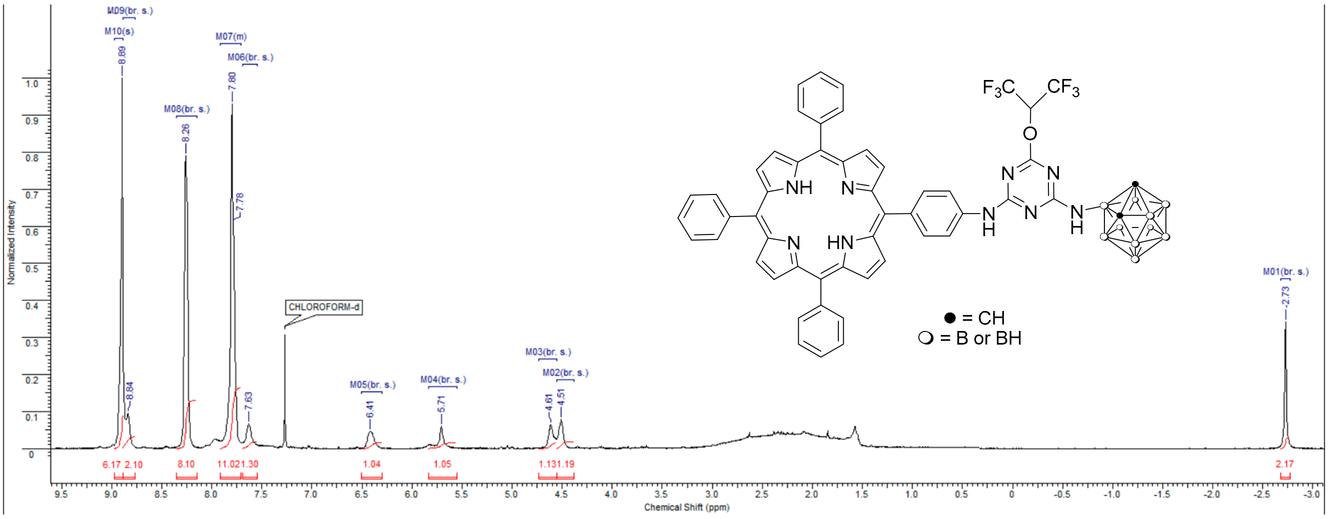

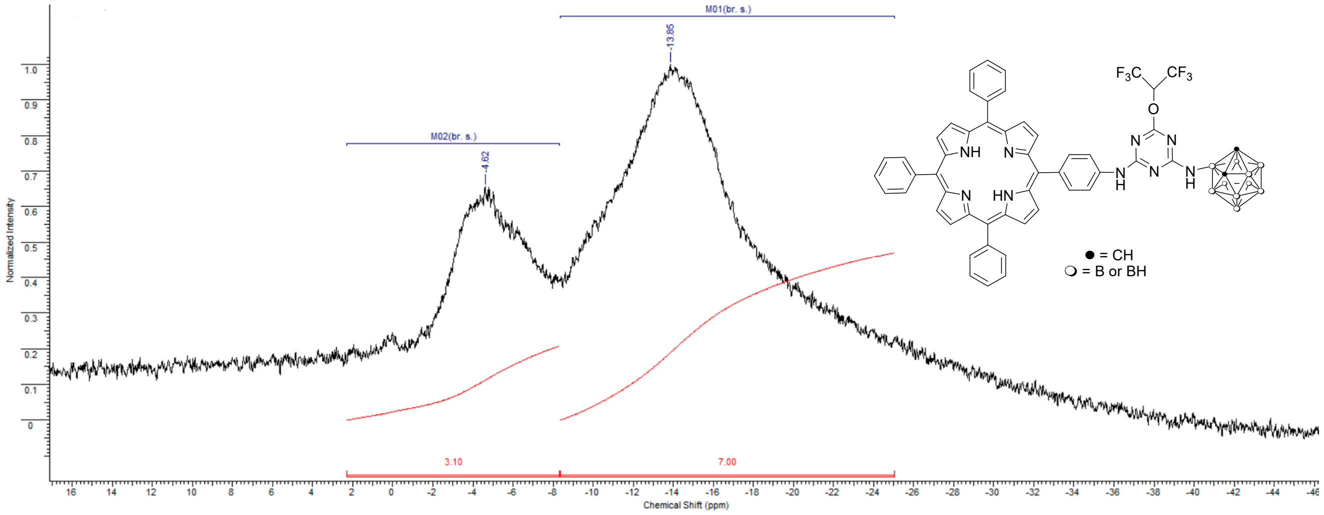

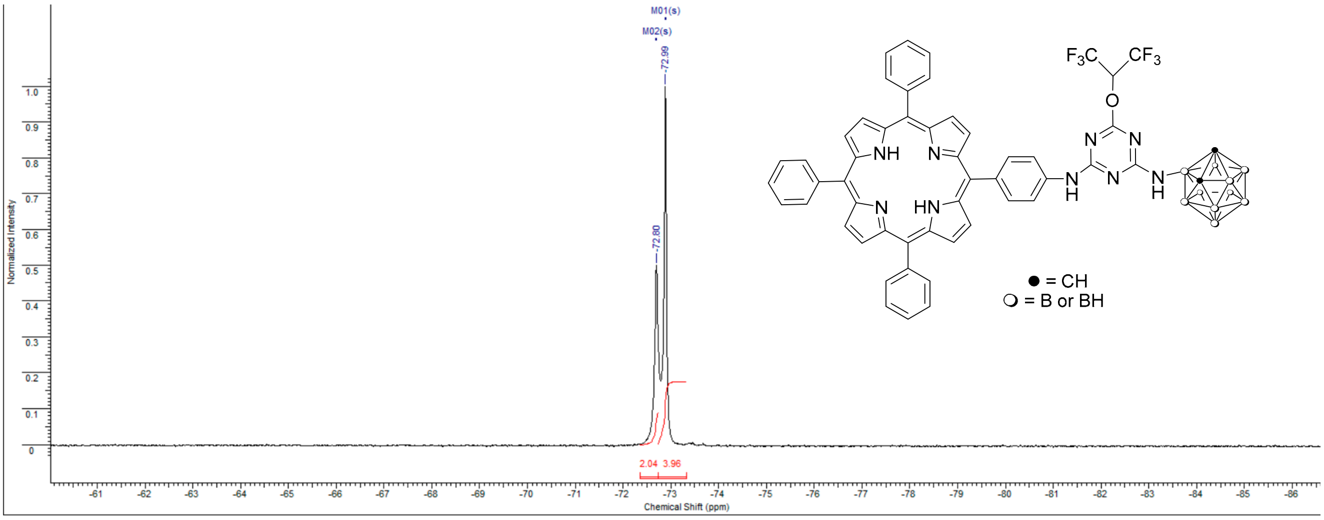

Figure S10. The ^{1}H, ^{11}B and ^{19}F spectra of **20** in $CDCl_3$.

11. The ^{1}H and ^{11}B NMR spectra of 5-{4-[[4-(*m*-carborane-9′-yl)thio]-[6-(aminoethylthio)]-(1,3,5)-triazine-2-yl]aminophenyl}-10,15,20-triphenylporphyrin (21)

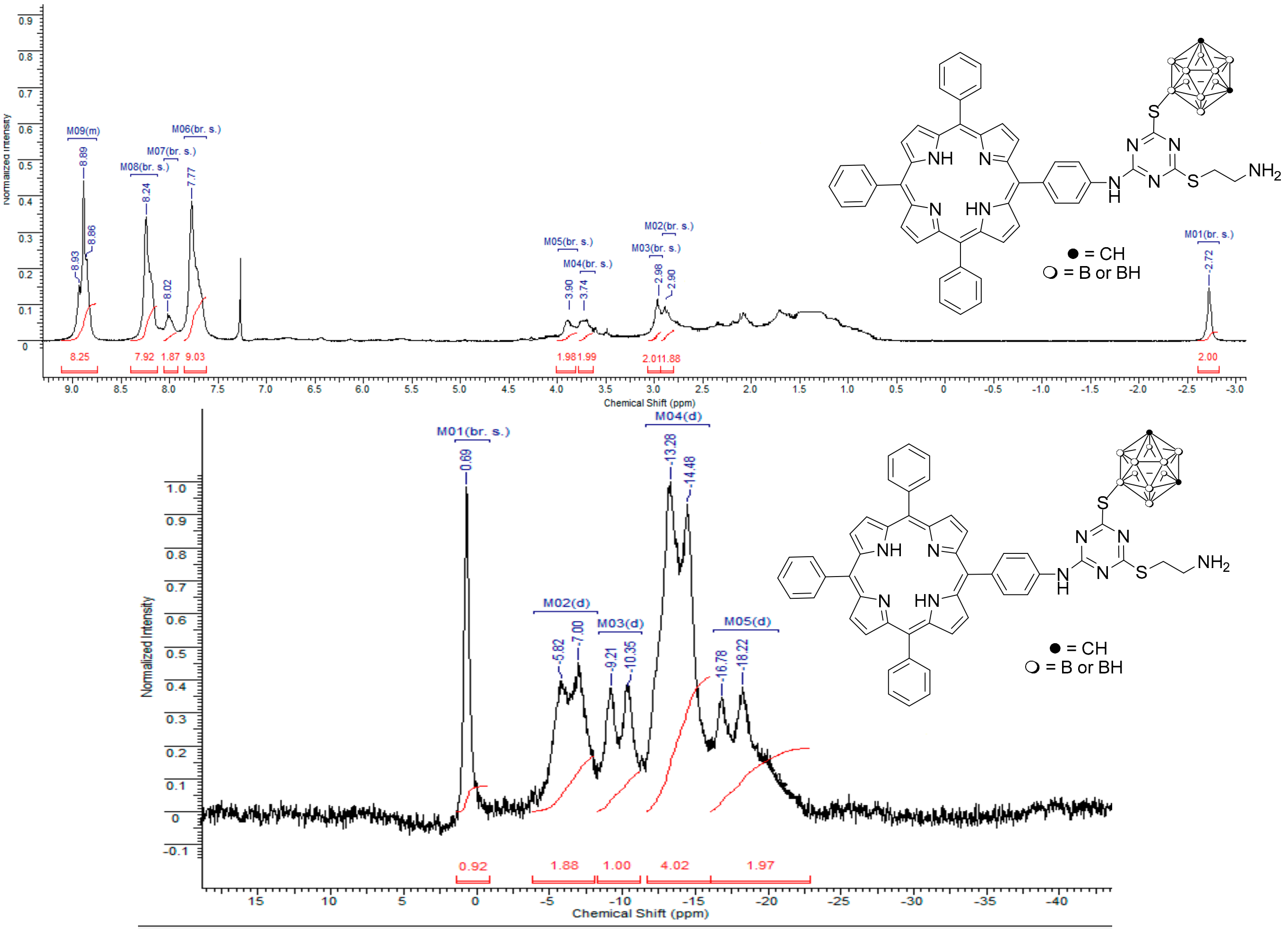

Figure S11. The ^{1}H, ^{11}B NMR spectra of **21** in $CDCl_3$.

12. The ^{1}H and ^{11}B NMR spectra of 5-{4-[[4,6-bis((4-(1-(*N*-(7,8-dicarbaundecaborate-3′-yl))maleimido)aminophenyl)-amino]-(1,3,5)-triazine-2-yl]aminophenyl}-10,15,20-triphenylporphyrin (24)

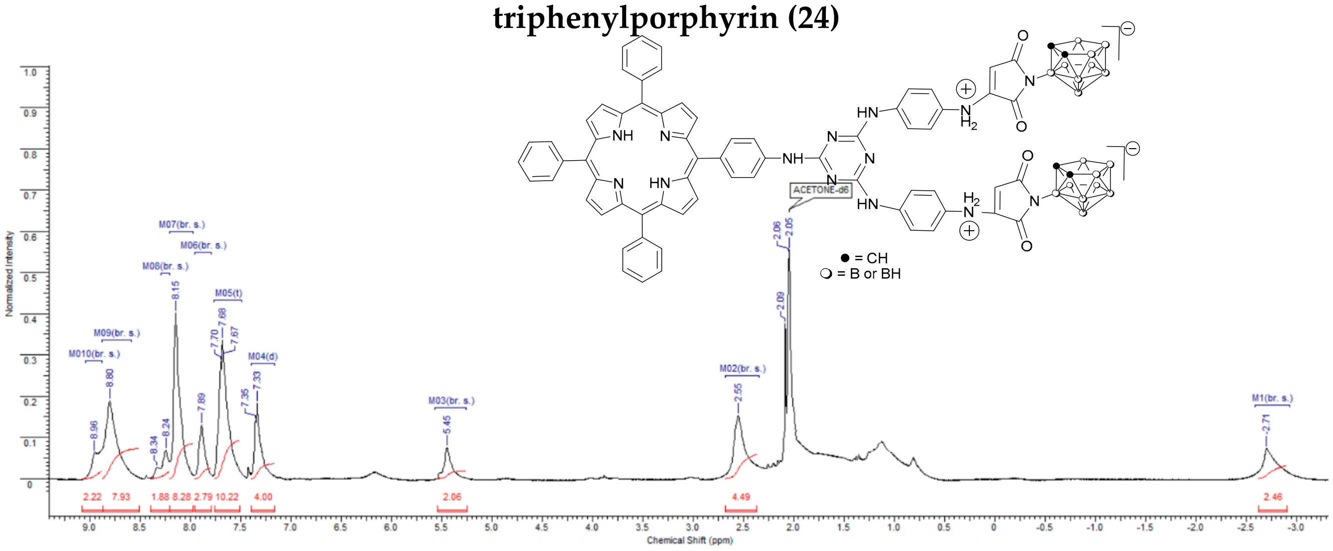

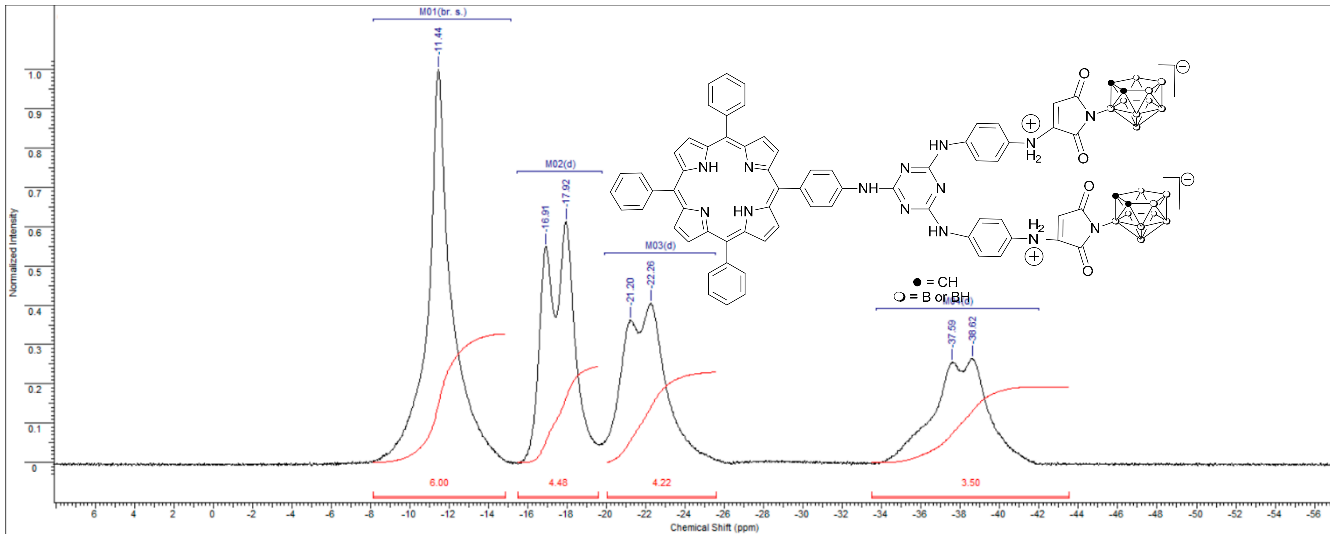

Figure S12. The ^{1}H and ^{11}B NMR spectra of **24** in $(CD_3)_2CO$.

13. The ^{1}H and ^{11}B NMR spectra of 5-{4-[[4,6-bis((4-(1-(*N*-(*o*-carborane-3′-yl))maleimido)aminophenyl)amino]-(1,3,5)-triazine-2-yl]aminophenyl}-10,15,20-triphenylporphyrin (25)

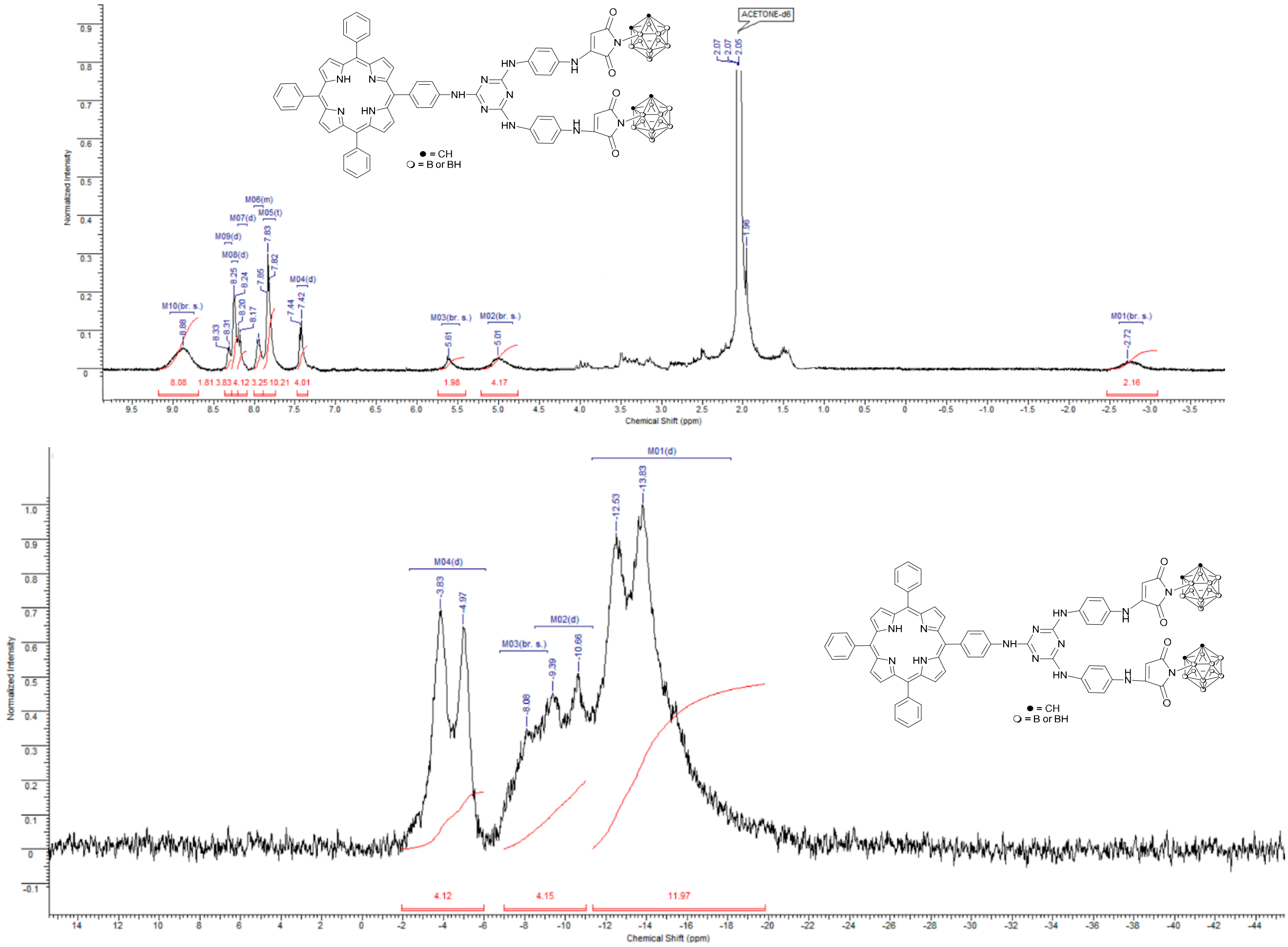

Figure S13. The ^{1}H and ^{11}B NMR spectra of **25** in $(CD_3)_2CO$.

14. The dark cytotoxicity of carboranylporphyrins 8, 9, 17, 18 and 24

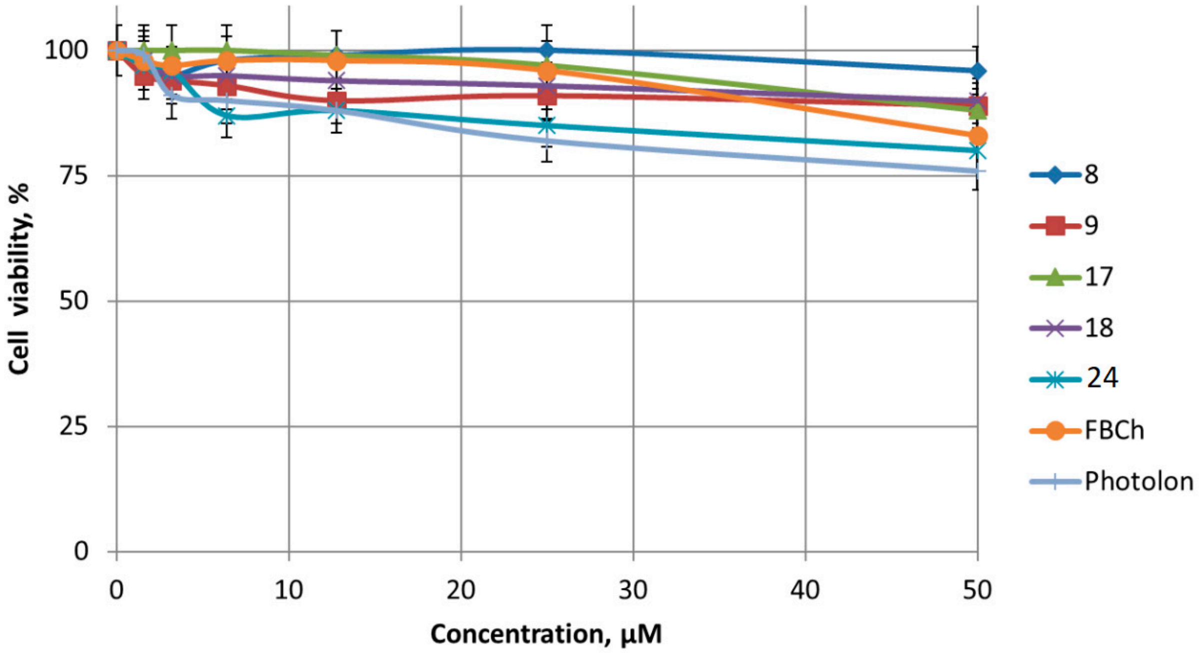

Figure S14. Dark cytotoxicity of compounds **8**, **9**, **17**, **18** and **24**.

15. The photoinduced cytotoxicity of carboranylporphyrins 8, 9, 17, 18 and 24

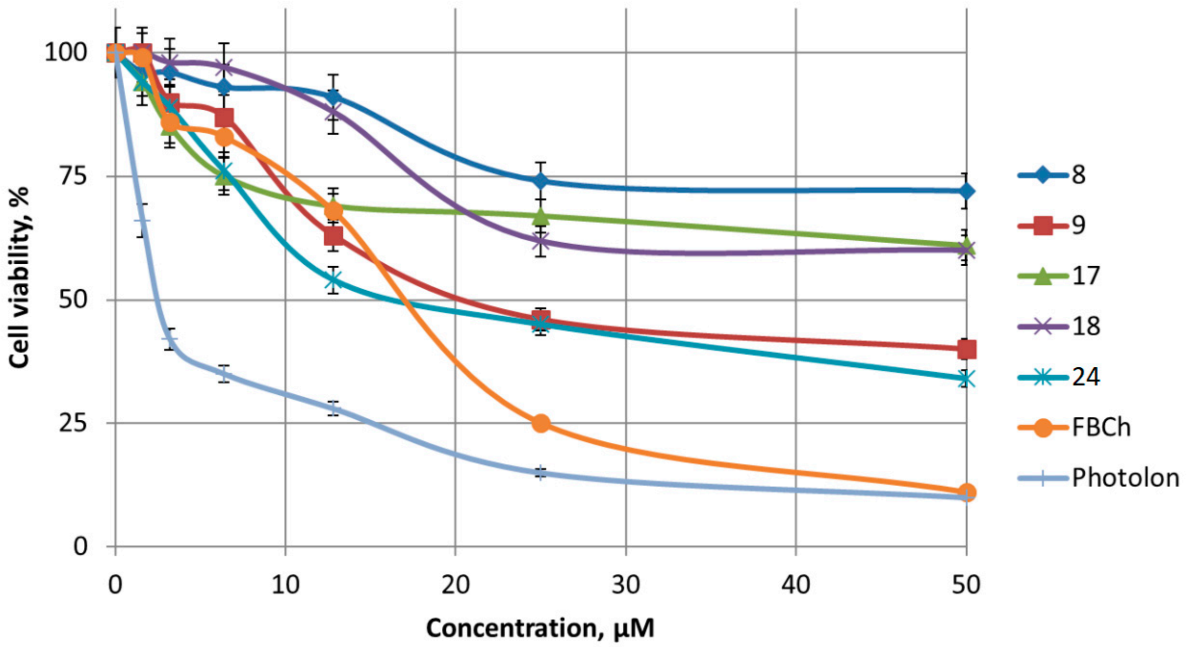

Figure S15. Photoinduced cytotoxicity of compounds **8**, **9**, **17**, **18** and **24**.
